# A computational model of neurodegeneration in Alzheimer’s disease

**DOI:** 10.1038/s41467-022-29047-4

**Published:** 2022-03-28

**Authors:** D. Jones, V. Lowe, J. Graff-Radford, H. Botha, L. Barnard, D. Wiepert, M. C. Murphy, M. Murray, M. Senjem, J. Gunter, H. Wiste, B. Boeve, D. Knopman, R. Petersen, C. Jack

**Affiliations:** 1 Department of Neurology, Mayo Clinic, Rochester, MN 55905 USA; 2 Department of Radiology, Mayo Clinic, Rochester, MN 55905 USA; 3 Department of Neuroscience, Mayo Clinic, Jacksonville, FL 32224 USA; 4 Department of Information Technology, Mayo Clinic, Rochester, MN 55905 USA; 5 Department of Health Sciences Research, Mayo Clinic, Rochester, MN 55905 USA

**Keywords:** Cognitive neuroscience, Alzheimer's disease

## Abstract

Disruption of mental functions in Alzheimer’s disease (AD) and related disorders is accompanied by selective degeneration of brain regions. These regions comprise large-scale ensembles of cells organized into systems for mental functioning, however the relationship between clinical symptoms of dementia, patterns of neurodegeneration, and functional systems is not clear. Here we present a model of the association between dementia symptoms and degenerative brain anatomy using F18-fluorodeoxyglucose PET and dimensionality reduction techniques in two cohorts of patients with AD. This reflected a simple information processing-based functional description of macroscale brain anatomy which we link to AD physiology, functional networks, and mental abilities. We further apply the model to normal aging and seven degenerative diseases of mental functions. We propose a global information processing model for mental functions that links neuroanatomy, cognitive neuroscience and clinical neurology.

## Abstract (web summary)

Low-dimensional representations of functional brain anatomy relevant for dementia syndromes may exist. Here the authors propose a computational model of mental functions to catalogue this anatomy in Alzheimer’s and related dementias.

## Introduction

Mapping biological functions to their anatomic substrates has been a central theme throughout medicine. At the core of the clinical practice of neurology is the localization of a particular clinical deficit to an anatomic substrate in the nervous system. Localizing limb strength to a lesion in the nervous system is usually straightforward, but in neurodegenerative disorders of the brain that cause dementia, clinical symptoms manifest as selective impairments in mental functions. Cognitive psychology describes these mental abilities using terms such as perception, emotion, memory, social cognition, language, and executive function. Clinical localization of these functions is poorly understood as there is no widely used model in neurologic practice describing the high-level relationships between anatomy, brain dynamics, and mental functioning to guide the clinical approach to these common conditions. This has led to recent calls to revise the psychological ontology using data driven methods^1^. Lack of understanding of this mental biology in terms of functions performed by brain networks also precludes the development of disease models that include physiology related to functional brain systems like the default mode network (DMN)^2–6^. To bridge this divide, mapping between concepts in clinical neuropsychology, neurology, and computational neuroscience is required.

From a computational neuroscience perspective, the diverse cognitive functions degraded by Alzheimer’s disease (AD) and related disorders, are conceptualized as emerging from the integration of ongoing microscale and mesoscale dynamic functional operations occurring within a relatively fixed spatial anatomy. In this respect, high-level mental abilities emerge from the computations performed from dynamic global integration of local integrators at these micro- and mesoscales^7^. These globally integrated units, or large-scale ensembles of coordinated neuronal activity, can be modeled as large-scale network topologies embedded in hierarchical adaptive network architecture^8^. These global network models can be decomposed into bigraphs representing instantaneous brain states that dynamically integrate over time to form the commonly observed static functional network architectures^5,9^. In this framework, properties of particular topologies are associated with specific classes of mental abilities. Therefore, a systematic spatial mapping of these network configurations may provide a model of the brain networks associated with dynamic optimization of perception, cognition, and behavior^5,9–11^. These networks, and mental abilities, are associated with neurodegenerative diseases of the brain^12^. Given that neurodegenerative diseases are functionally structured clinically and anatomically^13^, they encode a clinically relevant mapping between brain function and structure. Regional approaches to this clinical brain-behavior relationship are being replaced by functional network approaches^12,14,15^. Indexing a large number of brain state configurations associated with clinically relevant symptoms in perceptual, cognitive, and behavioral functions seems like an intractable problem on the surface due to the high dimensional nature of these configurations, but functional network topologies can also be described using a low-dimensional manifold^10,16,17^. This means that brain state configurations can be represented in a comparatively low dimensional space, such that any particular brain state can be largely characterized by a vector in this space. Neurotransmitter-modifiable activity within this manifold may be associated with diverse mental abilities^10^. Low dimensional principles are commonly utilized in movement neuroscience^18^. However, many computational operations relevant for movement neuroscience are at a different functional level relative to mental operations relevant for clinical neurodegenerative syndromes (e.g., perception, cognition, and behavior). In these syndromes, the level of functioning is clinically indexed by global scales such as the Clinical Dementia Rating (CDR) global score^19^, Global Assessment of Functioning (GAF)^20^, and/or global cognitive domain scores^21^. Therefore, the low dimensional representation of degenerative brain state configurations at this scale represent features of this global level of mental functioning.

Trajectories in a continuous manifold^10^, or sequence of binary states in a discrete manifold^5,9^, may be used to model network topologies associated with high-level mental abilities. Rather than relating impairment in a particular class of mental functions to a brain region as is commonly done in clinical practice, this model could associate clinical symptoms to altered dynamics in a portion of the manifold associated with that function. Disruption of a portion of the manifold may be characteristically associated with a particular dementia syndrome. In this context, previously observed altered dynamics in disease states^5^ may help characterize the similarity between brain atrophy and patterns of decreased^12^ functional connectivity that co-occur with increases in functional connectivity distant from atrophy^22,23^. In the current study we examine these relationships, and incorporate them into a model linking neurodegenerative anatomy, functional systems, and clinical symptoms. This is accomplished within a low dimensional framework that emphasizes functional modes of degeneration. This proposed framework is a requirement of complex systems models of AD, such as the cascading network failure model that relates dynamic spatial and temporal patterns in amyloid and tau accumulation to large-scale functional network dynamics^22,24–26^.

The neurobiology that allows for AD and related disorders to selectively target particular mental abilities, brain networks, or brain regions while sparing others, is unknown^27^. There are prominent individual differences in this selectivity leading to variable cognitive symptoms among types of AD dementia^28^, such as the typical late life amnestic syndrome and the younger-onset visual or dysexecutive variants^29^, resulting in a “paradox of syndromic diversity”^30^. Characterizing the factors related to this paradox, and individual variability in general, should inform the underlying neurobiology driving syndromic variability^31^. Recent investigations of individuals with typical amnestic AD and those with the visual variant of AD showed that they were indistinguishable at the molecular level^32,33^, but they can be distinguished at the network level^34,35^ or by tau spatial patterns that resemble the anatomy of functional brain networks^24^. This suggests that inter-individual variability in mental abilities affected by AD is partly driven by inter-individual differences in the macroscale functional pathophysiology of AD, as opposed to purely at the molecular level. This would be consistent with theories of AD pathogenesis that implicate large-scale network dynamics in disease pathogenesis alongside microscale misfolding of proteins^22,24–26^ that is in-line with general theories of network dysfunction in neuropsychiatric disease^36^. To test and improve upon AD models that include large-scale network physiology, a more complete model of the physiology of mental abilities and their relationship to brain networks and neuroanatomy is essential. This requires a model that spans computational neuroscience, clinical neuropsychology, and neurology, however this would require evidence for selective degeneration of modes/regions within the previously described manifold. It is also uncertain how such a model would relate to currently used clinical frameworks for classifying degenerative dementia syndromes using terms such as semantic dementia, primary progressive aphasia, progressive dysexecutive syndrome, behavioral variant of frontotemporal dementia, amnestic predominant dementia, and related terms.

In this model of neurodegeneration, a selective functional impairment seen in an individual with AD can be modeled as impaired dynamics in a particular portion of the manifold, or degenerative dynamics within a functional mode of operation^5^. In other words, the specific pattern of global dysfunction in an individual is represented by a particular parameterization of disease pathophysiology within this framework. In this computational disease model, individuals with neurodegenerative diseases represent “lesion studies” of functional modes associated with higher mental functions, as opposed to discrete regions or networks. We used this data to inform our model linking neurodegenerative diseases with brain function. We hypothesized that the inter-individual differences in neurodegeneration across the AD spectrum could be represented by a low-dimensional manifold that captures key features of our computational model of neurodegeneration. This has the potential to link AD pathophysiology and functional brain organization with the computational concepts in our model. The manifold identified in patterns of neurodegeneration may also be linked to the functional imaging literature and be aligned with mental symptoms observed in specific dementia syndromes. Therefore, this study attempts to link low-dimensional patterns of neurodegeneration to the existing neuroscience literature describing gradients of functional connectivity^37^, task activation patterns^38^, a variety of AD biomarkers, brain aging, and distinct clinical syndromes that selectively impair cognitive functions.

In this study, we report a low-dimensional representation of neurodegeneration and characterize its relationship to fundamental features of AD, linking it to the neuroscience literature and clinical syndromes related to brain function. This is accomplished through four main investigations: (1) patient data (*N* = 423) is used to derive the low-dimensional manifold via a latent space representation of glucose uptake across the AD clinical spectrum, (2) mental functions are mapped to the observed manifold using a functional meta-analysis and compared to functional connectivity data, (3) application and external validation of the predictive ability of the observed manifold in a large multi-site study (*N* = 410), and (4) additional clinical construct validation of the functional-anatomic mapping by projecting data from normal aging (*N* = 1121) and clinically defined dementia syndromes (*N* = 291) selectively targeting memory, executive functions, language, behavior, movement, perception, semantic knowledge, and visuospatial abilities. The first 10 dimensions of this low-dimensional representation explained 51% of the variance in glucose uptake. The anatomic patterns of this representation are related to gradients of functional connectivity and encode a mapping of meta-analytic functional task activation patterns. The eigenvalues of this manifold are predictive of markers of AD within the cohort and validated in an external sample. Within our theoretical framework, these observations are consistent with a global information processing model of impaired mental functions in dementia syndromes. This hypothetical computational construct was consistent with the known brain-behavior relationships observed in normal aging and seven dementia syndromes.

## Results

### Patients

To ensure that global information processing was disrupted in the individuals included in our investigation, we selected patients with evidence of clinically relevant cognitive impairment using a clinical dementia scale, defined here as a CDR global score greater than zero. In this patient population, we aimed to investigate brain physiology that would be sensitive to degeneration of brain function that can be reliably measured and etiologically non-specific. Therefore, we studied glucose uptake measured by F18-fluorodeoxyglucose (FDG) positron emission tomography (PET), a widely used functional imaging modality in routine use in our clinical practice currently. In the current research framework for AD, FDG-PET is considered a biomarker of neurodegeneration^39^, therefore in this selected population the majority of individual variation in FDG-PET uptake would be related to a neurodegenerative etiology. We further limited our FDG-PET analysis to individuals who had evidence of microscale AD pathophysiology (i.e., elevated beta-amyloid PET) making this an analysis of AD associated neurodegeneration that manifests in individual differences in altered glucose uptake. While this focuses our investigation to individuals with a microscale element of AD pathophysiology by definition^39^, it does not preclude other co-morbid conditions and therefore allows for a sampling of the complete spectrum of beta-amyloid associated cognitive impairment. We identified 423 patients that met these inclusion criteria (Table 1). The characteristics of the validation cohort from the Alzheimer’s Disease Neuroimaging Initiative (ADNI) are also listed in Table 1.

**Table 1:** Mayo and Alzheimer’s disease neuroimaging initiative sample characteristics.

	Mayo	ADNI	*P*-value
*N*	423	410	–
Age (median [Q1,Q3])	77.4 [69.1, 83.5]	74.5 [69.6, 79.4]	0.001
Male (%)	244 (57.7)	223 (54.4)	0.375
Education (median [Q1,Q3])	15 [12,17]	16 [14,18]	<0.001
*APOE e4*+ (%)	243 (61.7)	283 (69.2)	0.030
CDR (%)	–	–	<0.001
0.5	274 (64.8)	310 (75.6)	–
1	106 (25.1)	96 (23.4)	–
2	39 (9.2)	4 (1.0)	–
3	4 (0.9)	0 (0.0)	–
CDR-SOB (median [Q1,Q3])	3.0 [1.0, 5.5]	2.0 [1.0, 4.4]	0.004
MMSE (median [Q1,Q3])	24 [21,27]	26 [24,28]	<0.001
FDG (median [Q1,Q3])	1.25 [1.09, 1.40]	1.16 [1.05, 1.28]	<0.001

Source data are provided as a Source Data file.

*APOE e4+* carriage of an APOE-ε4 allele, *CDR* Clinical Dementia Rating Scale, *CDR-SOB* Clinical Dementia Rating Scale Sum of Boxes, *MMSE* Mini-Mental State Examination.

### Low-dimensional representation of neurodegenerative anatomy

Individual variability in patterns of glucose uptake in these patients are a parameterization of amyloid-associated degenerative AD neurobiology^31^. We decoded this parametrized AD pathophysiology by performing principal component analysis within a flexible framework we refer to as Between-subject variability Projection and Reduction (BPR) that emphasizes the importance of the component parts of the analysis related to sample selection and patient factors for representing a pathophysiology of interest driving the observed variability. We explored different elements in the BPR framework (e.g., subject selection, data preprocessing, and dimensionality reduction method) and these are discussed in more detail in the Supplementary Methods (Supplementary Figs. 1–7).

**Fig. 1:**
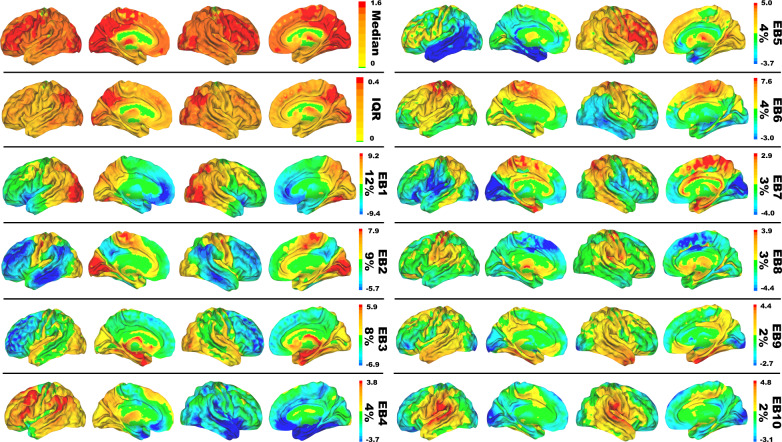
Surface renderings of glucose eigenbrains. Eigenbrain decomposition of glucose uptake in 423 Alzheimer’s patients reveals a low-dimensional set of large-scale patterns that explain 51% of the variance among patients. Surface renderings of median, interquartile range (IQR), and eigenbrain (EB) intensities for the first 10 eigenbrains are displayed. The percentage of variance explained by each is listed to the right of the color bar of arbitrary units.

The biologically motivated BPR framework is able to incorporate many commonly used analytic techniques to identify patterns characterized by between subject covariance. As we applied it to our imaging data using principal component analysis, it is a three-dimensional computational equivalent of the two-dimensional eigenfaces facial recognition algorithm as implemented by Turk and Pentland for defining a face space^40^. Unsupervised linear (singular value decomposition) and non-linear (Laplacian eigenmaps) methods for the manifold decoding step performed similarly in our data suggesting that the linear solution is a good approximation of the manifold. In contrast, a full sampling of the parameterization of the manifold of interest, via between subject variances, is required to replicate the same low-dimensional representation. This is because BPR, and related analyses, of FDG-PET images from a disease class will index meaningful features of altered glucose uptake caused by the pathophysiologic process of interest in the patient population being studied. In the population we studied, this algorithm produced a low dimensional linear basis-set of eigenbrains or EBs (Fig. 1), that describes 51% of the variability in the FDG images. These EBs describe modes of variation in glucose uptake among the group that index meaningful functional brain properties relevant to AD biology (Table 2). To support our main hypothesis that this biologically meaningful latent space reflects aspects of our computational model of neurodegeneration, we conducted a series of experiments linking this latent space to existing neuroscience literature and clinical syndromes related to large-scale brain function. These analyses support our hypothesis that these degenerative patterns can be associated with computational principles in our model. In this computational model of mental functions, the observed low-dimensional representation of neurodegeneration is interpreted as quantifying latent parameters within the manifold.

**Table 2:** Predictive models in the mayo cohort for key effects of Alzheimer’s disease.

Variable	*N*	*R*^2^	_adj_*R*^2^	_pre_*R*^2^	EB1	EB2	EB3	EB4	EB5	EB6	EB7	EB8	EB9	EB10	*P*-value	_adj_*P*-value
FDG_AD_	423	0.74	0.73	0.72	0.39	−0.64	−0.03	−0.01	0.11	−0.05	−0.23	−0.20	0.26	−0.02	2.2E-16	2.2E-15
Age	423	0.66	0.65	0.64	0.54	−0.33	−0.32	0.06	−0.18	−0.13	0.23	0.17	−0.09	0.12	2.2E-16	2.2E-15
FDG_HS_	423	0.63	0.62	0.60	0.52	−0.40	−0.29	0.10	0.10	−0.01	−0.12	−0.22	0.11	0.14	2.2E-16	2.2E-15
MMSE	403	0.57	0.56	0.54	0.24	−0.60	0.06	0.15	−0.17	−0.03	−0.05	−0.09	0.32	−0.01	2.2E-16	2.2E-15
MRI_THK_	417	0.51	0.50	0.48	0.09	−0.50	0.27	−0.18	−0.05	−0.02	−0.17	−0.17	0.31	−0.02	2.2E-16	2.2E-15
Braak NFT	67	0.60	0.52	0.42	−0.12	0.25	−0.04	0.43	0.58	0.04	−0.31	−0.40	−0.16	0.08	7.2E-08	7.2E-07
CDR-SOB	423	0.45	0.44	0.42	−0.18	0.54	0.01	−0.06	0.09	0.08	0.05	0.12	−0.31	0.00	2.2E-16	2.2E-15
Tau-PET	138	0.51	0.47	0.41	−0.29	0.48	0.09	0.08	0.21	0.03	−0.28	−0.02	−0.20	0.08	9.5E-16	9.5E-15
Hippo Vol	417	0.40	0.39	0.37	−0.23	−0.17	0.34	−0.18	−0.04	0.05	−0.07	−0.11	0.34	−0.21	2.2E-16	2.2E-15
CDR	423	0.34	0.33	0.30	−0.17	0.47	0.04	−0.05	0.08	0.08	0.08	0.07	−0.26	−0.01	2.2E-16	2.2E-15
Sex	423	0.21	0.20	0.17	0.09	−0.11	0.09	0.02	0.01	0.19	0.03	0.31	0.07	−0.22	2.2E-16	2.2E-15
UDPRS	407	0.20	0.18	0.16	−0.13	0.04	0.17	−0.13	−0.25	0.04	0.23	0.15	0.03	−0.02	5.3E-15	5.3E-14
RBD	246	0.20	0.17	0.13	−0.17	−0.09	0.17	−0.08	−0.16	0.11	0.21	0.19	−0.01	−0.12	3.9E-08	3.9E-07
FDG_DLB_	423	0.16	0.14	0.12	−0.26	−0.12	0.23	0.06	−0.04	−0.11	0.05	−0.02	0.01	−0.09	7.5E-12	7.5E-11
Amyloid-PET	423	0.16	0.14	0.12	0.02	0.11	−0.23	0.05	0.12	−0.11	−0.10	0.08	−0.23	0.03	7.6E-12	7.6E-11
Duration	118	0.25	0.18	0.10	−0.14	0.18	−0.26	−0.03	0.17	−0.04	−0.13	0.06	−0.25	0.09	3.6E-04	3.6E-03
Lewy path	64	0.39	0.27	0.06	−0.22	0.20	−0.02	−0.17	−0.10	−0.16	0.35	0.51	0.04	0.08	1.8E-03	1.8E-02
E4+	384	0.08	0.06	0.03	0.04	0.09	0.02	0.18	0.14	0.13	−0.03	−0.07	−0.03	0.01	1.9E-04	1.9E-03

For each of the dependent variables, the first 10 eigenvalues were used as predictors in a multivariate linear regression model. The R^2^, adjusted R^2^, predicted R^2^, standardized beta coefficients, two-sided p-value, and multiple comparisons adjusted *p*-values using the Bonferroni correction are displayed for each model. Source data are provided as a Source Data file.

*FDG*_*AD*_ FDG SUVR in AD signature regions, *FDG*_*HS*_FDG SUVR in hippocampal sclerosis signature regions, *FDG*_*DLB*_FDG SUVR in DLB signature regions, *MMSE* Mini-Mental State Examination, *MRI*_*THK*_ MRI thickness in AD signature regions, *Braak NFT* Braak neurofibrillary tangle stage, *CDR*Clinical Dementia Rating Scale, *CDR-SOB* Clinical Dementia Rating Scale Sum of Boxes, *Hippo Vol* Hippocampal volume, *UPDRS* Unified Parkinson’s Disease Rating Scale, *RBD* REM sleep behavior disorder, *Duration* disease duration, *E4+* carriage of an APOE-ε4 allele.

### Functional mapping of the anatomy described by the glucose eigenbrains

**Fig. 2:**
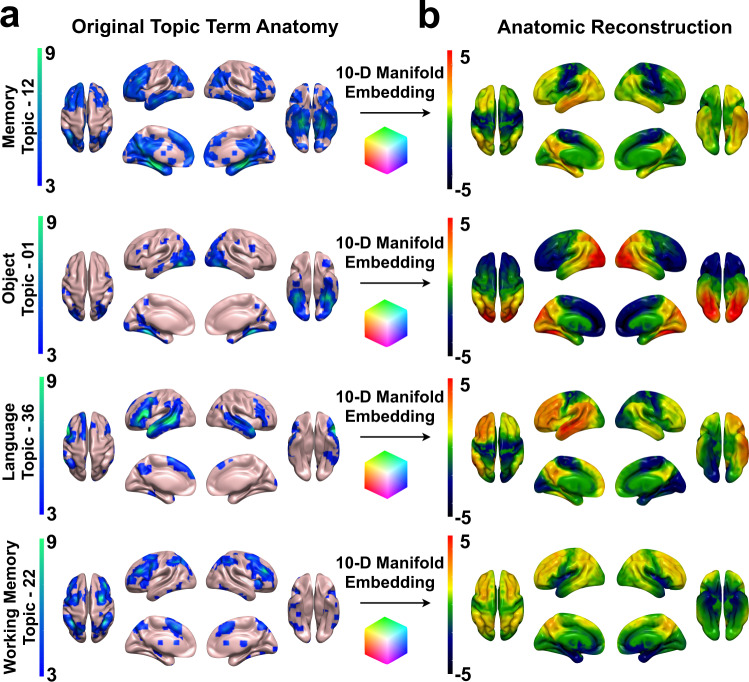
Embedding large-scale brain patterns associated with mental function topics. **a** Neurosynth (http://neurosynth.org/analyses/topics/) anatomic maps (*z*-scores) for four topics related to AD clinical syndromes are displayed on surface renderings. **b** The manifold embeddings (arbitrary units) of these topics were used to generate the anatomy associated with that point in the manifold and projected onto surface renderings. This demonstrates faithful representation of these anatomic patterns associated with mental topics in the manifold coordinate system (also see Fig. 6 and Supplementary Fig. 8).

**Fig. 3:**
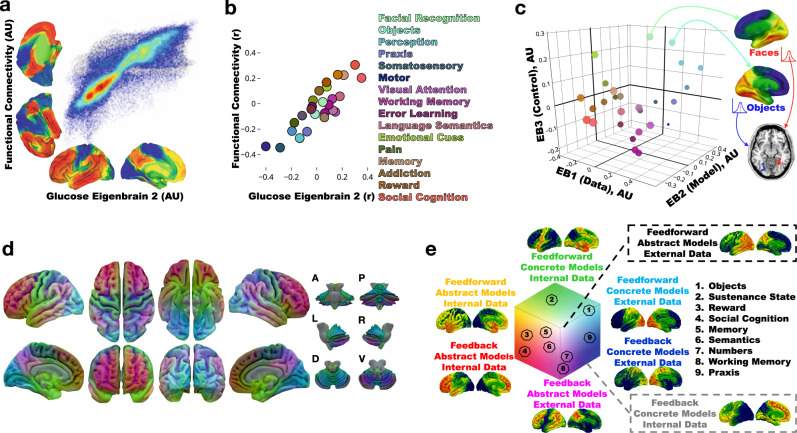
Global information processing interpretation of low-dimensional degenerative patterns in dementia. **a** Joint histogram between the principal axes of functional connectivity^37^ and glucose EB2. **b** Neurosynth decoding of the principal axes of functional connectivity versus glucose EB2 decoding. Select topic terms are color-coded on the right (color coding is the same as in **c**). **c** Scatter plot of topic term decoding for glucose EB1-3. Source data are provided in Supplementary Table 1. For the color-coding, each EB decoding was used as a RGB channel (EB1 = Blue, inverted polarity EB2 = Red, EB3 = Green). Radius of the points encodes depth along EB2. Generated anatomy using the 10-D EB decoded coordinates for faces and objects are displayed on the right and left hemisphere (respectively) surface renderings. Below these, axial brain slice with the peak voxels from the faces and objects anatomic projection overlaid highlighting regions near the fusiform face area (red) and visual word form area (blue) respectively encoded at these points. **d** The same RGB color-coding was applied in a voxel-wise manner using the intensities from EB1-3 producing a continuous functional parcellation of brain anatomy along these gradients. **e** The global information processing state space representation of the same color mapping with the approximate location of nine cognitive topic terms from panel **c** overlaid and numbered. A surface rendering of the anatomic correlates, generated from linear combinations of EB1-3 weighted by the position in state space, for the eight extremes of the cube are displayed near the portion of state space represented. AU-arbitrary units; A-anterior; P-posterior; L-left lateral; R-right lateral; D-dorsal; V-ventral.

**Fig. 4:**
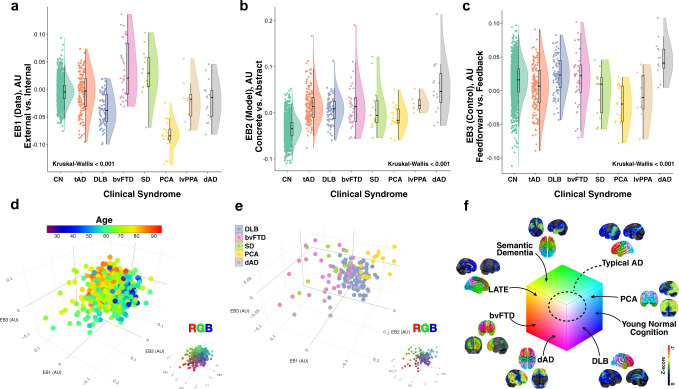
Glucose eigenbrains across normal aging and seven clinical dementia syndromes. Rain cloud plots^61^ with data distribution and jittered raw data points for biologically independent unique patient observations on either side of boxplots (horizontal lines denote median values; boxes extend from the 25th to the 75th percentile of each group’s distribution of values; vertical extending lines denote values within 1.5 interquartile range of the 25th and 75th percentile of each group) of eigenvalues for **a** EB1, **b** EB2, and **c** EB3 for cognitively normal amyloid negative individuals across the aging spectrum (CN, *n* = 1121), typical AD (tAD, *n* = 137), dementia with Lewy bodies (DLB, *n* = 72), behavioral variant of frontotemporal dementia (bvFTD, *n* = 33), sematic dementia (SD, *n* = 11), posterior cortical atrophy (PCA, *n* = 15), logopenic variant of primary progressive aphasia (lvPPA, *n* = 8), and dysexecutive AD (dAD, *n* = 15). Source data are provided as a Source Data file. **d** Scatter plot for the first 3 EBs for all these subjects with age color mapping showing the youngest individuals (blue) at the opposite extreme from the oldest individuals (red). The same plot with RGB color mapping for reference to other figures is inset in the bottom right. **e** Scatter plot for the first 3 EBs for 5 dementia syndromes highlighting the differential mapping across the manifold with clinical syndromes coinciding with predictions made by the functional mapping in Fig. 3. The same plot with RGB color mapping for reference to other figures is inset in the bottom right. **f** The same RGB color mapping used in Fig. 3 indicating the anatomy and state space location for each clinical group (including an example of limbic-predominant age-related TDP-43 encephalopathy [LATE]^46^). A representative single subject clinical FDG-PET (Cortex ID, GE Healthcare, Chicago, IL, USA) with *z*-scores relative to age matched controls color-coding the degree of hypometabolism for one patient from each group is also displayed. AU-arbitrary units.

We used a Neurosynth (www.neurosynth.org)^41^ functional topic terms^38^ based decoding^42^ as a common framework to compare the functional anatomy captured in this study to the existing functional MRI literature in a similar manner as Shine et al.^10^ and Margulies et. al.^37^, allowing for a common understanding of these diverse findings in the same meta-analytic functional terminology. The functional topic term decoding for a single topic across all 10 EBs can also be used as an embedding of that topic in our model’s coordinate system. The coordinates of that embedding can then be used as EB weights in a linear anatomical reconstruction of that functional topic (Fig. 2). The linear combination of the smooth gradients described by the EBs produce whole brain patterns associated with each functional topic. Peak values in these reconstructed maps correspond to regions of peak activation associated with brain patterns observed during performance of these tasks. These topic term embeddings can also be used as ‘functional waypoints’ to aid in interpreting functional correlates of large-scale anatomic patterns of disruption in patients. To do this on a single subject level across functional topic terms, we linked values in our model’s coordinate system continuously to the functional imaging literature via a full topic term decoding of each EB (Fig. 3) and then embedded individual subjects into this well characterized functional-anatomic coordinate-based model (Fig. 4).

In this cohort, the first three EBs account for 29% of the variance and are related to hemispherically symmetric orthogonal axes of brain function that capture the majority of the manifold. Therefore, we focused on presenting the results for characterizing these three EBs. The functional axis captured in EB2 (Fig. 3a) was nearly identical to the principal gradient defined by Margulies et al.^37^ using functional connectivity data from cognitively unimpaired individuals. The meta-analytic functional topic terms-based decoding for EB2 and the same decoding of the principal gradient were highly correlated (Fig. 3b). This EB fully indexes the glucose uptake in the principal gradient of macroscale cortical organization, characterized at one extreme by heteromodal association cortex (centered on DMN regions) and on the other extreme by primary sensory and motor regions. This fundamental organizing feature of brain function was first observed in FDG-PET^6^, subsequently identified in patterns of functional connectivity^2^, and also shown to be impaired in AD^3^. Features of this pattern (e.g., sparing of the sensorimotor strip) are also routinely used by clinicians when interpreting FDG scans from patients^43^. The fact that variation in glucose metabolism in AD takes place along this and other macroscale functional gradients is consistent with our hypothesis that AD can be modeled as altered flow through a low dimensional functional manifold that represents large-scale network configurations related to mental functions^10^.

This structural-functional mapping of the EBs can be compactly represented and visualized in a three-dimensional approximation of the low dimensional manifold using the first three eigenbrains. This can be done using a latent space coordinate system (Fig. 3c, e), or in anatomic space (Fig. 3d). The RGB color map of the anatomic representation demarcates functionally meaningful brain parcels based on the patterns of continuous variation in the gradients of the first three eigenbrains. This produces analogous results to defining brain parcels based on regional variation in cytoarchitectonics within an individual^44^, but was derived from variation in degenerative patterns across individuals.

Each of the axes, or latent variables in our model, can be conceptually simplified and dichotomized via axis polarity informed by this brain-behavior mapping (EB1: data source [internal vs. external], EB2: model form [abstract vs. concrete], and EB3: control type [feedback vs. feedforward]). These conceptual labels are hypothetical based on the relations between functional topic term mappings, anatomic connectivity, functional activation, and degenerative clinical symptoms described here.

The three-dimensional approximation is hemispherically symmetrical, but EB4 and EB5 can be included to capture breaks in symmetry and cumulatively explain 38% of the variance in the dataset. Naturally, the relative variance explained depends on the phenotypic composition of the cohort studied, in line with the BPR formulation. For example, EB5 captures hemispheric asymmetries in the left temporal lobe, including regions relevant for language functions, and eigenvalues were higher for the patients diagnosed with the language-variant of AD relative to the rest of the cohort, two-sided two-sample *t*(421) = 3.69, *p* < 0.001. The topic terms-based decoding of all 10 EBs and the principal gradient from Marguiles et. al.^37^ are presented in Table 3.

**Table 3:** Neurosynth topic term decoding of eigenbrains and principal gradient of functional connectivity.

Summary term	Topic term number	EB1	EB2	EB3	EB4	EB5	EB6	EB7	EB8	EB9	EB10	FC gradient
Langue comprehension	36	−0.02	−0.35	−0.04	0.19	−0.15	−0.03	−0.15	−0.12	0.04	0.13	0.20
Social	17	−0.15	−0.30	−0.05	−0.05	−0.01	−0.13	−0.05	−0.11	−0.04	−0.11	0.31
Memory	12	0.05	−0.21	0.08	0.06	−0.17	−0.16	−0.02	−0.18	0.06	−0.29	0.22
Language semantics	44	0.09	−0.18	−0.01	0.18	−0.21	0.01	−0.22	−0.12	0.04	0.07	0.06
Negative emotion	40	−0.38	−0.16	0.01	−0.22	−0.06	−0.16	−0.05	−0.02	−0.07	−0.17	0.23
Visual attention	41	0.35	−0.15	−0.05	0.00	0.11	0.04	−0.02	−0.10	−0.17	−0.04	−0.06
Language perception	20	0.22	−0.13	0.01	0.22	−0.12	0.01	−0.22	−0.09	0.00	0.17	−0.05
Numerical	42	0.21	−0.12	−0.10	0.08	0.08	0.01	0.15	−0.09	−0.01	−0.05	0.04
Working memory	22	0.14	−0.09	−0.20	0.13	0.06	0.08	0.10	−0.04	−0.04	−0.05	−0.01
Emotional cues	23	−0.28	−0.08	0.20	−0.24	−0.11	−0.20	−0.06	−0.02	−0.01	−0.09	0.13
Reward	29	−0.34	−0.08	−0.05	−0.10	0.04	−0.13	−0.07	0.08	−0.17	−0.23	0.17
Response preparation	47	0.15	−0.06	−0.22	0.04	0.14	0.22	0.03	−0.06	−0.13	0.00	−0.07
Hearing	32	−0.04	−0.06	0.03	0.05	0.00	0.06	−0.23	0.12	0.09	0.49	−0.11
Facial recognition	5	0.25	−0.05	0.29	−0.15	−0.09	−0.23	−0.10	−0.15	0.11	−0.09	0.01
Addiction	27	−0.27	−0.02	−0.02	−0.10	0.05	−0.09	0.06	0.05	−0.08	−0.17	0.10
Objects	1	0.40	0.01	0.27	0.01	−0.15	−0.09	−0.13	−0.15	0.10	−0.06	−0.08
Sustenance state	26	−0.30	0.03	0.06	−0.15	−0.03	−0.07	−0.07	0.14	−0.08	−0.07	0.03
Error learning	25	−0.01	0.04	−0.19	0.09	0.16	0.11	0.02	0.07	−0.08	0.02	−0.02
Response inhibition	8	−0.15	0.04	−0.13	−0.14	0.14	0.14	0.01	0.07	−0.15	0.02	−0.04
Praxis	0	0.38	0.08	0.02	0.07	0.06	0.26	0.00	0.01	0.03	0.21	−0.22
Stimulus response	19	0.00	0.12	−0.06	−0.01	0.11	0.04	−0.03	0.08	−0.11	−0.02	−0.09
Motion perception	11	0.57	0.13	0.17	−0.02	0.04	0.08	−0.05	−0.05	−0.04	0.06	−0.27
Perception	3	0.39	0.14	0.18	0.02	−0.02	0.10	−0.25	0.02	−0.02	0.20	−0.27
Pain	48	−0.35	0.18	−0.03	−0.11	0.12	0.12	−0.05	0.20	0.00	0.24	−0.15
Directed gaze	15	0.41	0.20	0.07	−0.01	0.09	0.15	0.08	−0.09	−0.02	−0.11	−0.22
Somatosensory	35	0.02	0.29	−0.04	0.00	0.17	0.35	0.06	0.22	0.05	0.38	−0.35
Motor	49	0.08	0.40	−0.17	0.15	0.16	0.38	0.07	0.14	0.07	0.30	−0.34

### Predictive modeling of factors related to AD

Together, the set of 10 EBs could be used to predict key demographic, imaging, clinical, and pathologic variables associated with the effects of AD (Table 2). In other words, indexing variation in glucose uptake in brain systems associated with mental functions and large-scale networks within a global information processing model is highly predictive of key effects of AD biology on an individual.

### External validation of predictive modeling

We next validate the predictive ability of quantifying dysfunction in our computational model in an independent cohort. Using the simple multivariate linear regression models from this cohort (Table 2) to predict the age of patients from an independent database (*N* = 410) available as part of the Alzheimer’s Disease Neuroimaging Initiative (Table 1), we achieved a mean absolute error of 5.1 years using a linear 10 EB model. Similar results were obtained predicting other variables in the dataset related to glucose uptake, cognition, and disease severity, with peak prediction performance achieved with models using 8–20 EBs (Supplementary Figs. 9 and 10). This predictive ability, across diverse variables using simple interpretable linear regression models, is evidence of the predicted association between our computational model and the expression of AD pathophysiology within an individual and serves as validation of our results in a multisite study. However, it should be noted that we did not attempt to optimize our manifold learning or predictive modeling for any particular predictive task in the current work, but demonstrate its potential to do so across diverse tasks relevant to neurodegeneration in a computational model relatable to functional connectivity gradients (Fig. 3), task activation patterns (Figs. 2 and 3), and clinical reasoning about degenerative brain conditions affecting perception, cognition, and behavior (Fig. 4).

### Clinical symptoms and the computational model of neurodegeneration

We embedded a large cohort of Mayo Clinic participants in our model’s representation using the eigenbrains derived from only the 423 individuals with amyloid-associated cognitive impairment (Fig. 4). See the Supplementary Methods for an exploration of the effect of cohort on eigenbrain definition (Supplementary Figs. 5 and 6).

This cohort included cognitively unimpaired individuals with negative amyloid-PET scans (*n* = 1121) across the age spectrum (median age [q1, q3] = 65 [57,74], range = 30–93) and seven clinically defined age-associated dementia syndromes: typical Alzheimer’s disease (tAD, *n* = 137), Dementia with Lewy Bodies (DLB, *n* = 72), behavioral variant of frontotemporal dementia (bvFTD, *n* = 33), semantic dementia (SD, *n* = 11), posterior cortical atrophy (PCA, *n* = 15), logopenic variant of primary progressive aphasia (lvPPA, *n* = 8), and dysexecutive Alzheimer’s disease (dAD, *n* = 15).

Each clinical syndrome could be characterized at the group level by their distribution along the first three coordinates of our manifold in a manner reflecting their distinguishing clinical features (Fig. 4a–c). Manifold learning that optimizes for group separation was not the goal of this analysis. Instead, we set out to observe the interpretable relationships between clinical symptoms that are characteristic of each phenotype and our model’s functional terminology derived from the association with functional connectivity and task activation patterns (Figs. 2 and 3). Consistent with clinical experience, and the fact that multiple pathologies are the most common pathologic findings in autopsy studies^45^, clinical phenotypes did not separate into distinct clusters in the first three dimensions of the model. Instead, they spread out along a continuum with distinct phenotypes collecting near the extremes (Fig. 4e, f). The relative location of the latent space embedding between phenotypes also reflects known shared pathologic similarities between clinical phenotypes (e.g., TDP-43 pathology in both late life amnestic dementia syndromes^46^ and semantic dementia or the co-occurrence of AD and DLB associated pathology^47^). Using a higher dimensional embedding and a multi-class classifier and/or optimizing manifold learning for group separation may improve clinical group separation, but that is not the goal of the current study as this may obscure a more generalizable and interpretable representation.

All the cognitive dementia syndromes differed from cognitive aging in terms of brain regions involved in abstract model formation (Fig. 4b). Using all 10 EBs as predictors in a logistic regression model with L2 penalty achieves the following performance on the task of predicting the presence or absence of clinical dementia (CDR global score greater than zero), averaged over 5-fold cross validation and with the estimated 95% confidence interval: 90.9 ± 2.6% accuracy, 89.7 ± 3.3% ROC AUC, 88.5 ± 6.6% precision, 68.1 ± 9.0% recall, and 0.769 ± 0.060 F1 score.

In our proposed framework for brain-behavior mapping, both PCA and DLB displayed characteristic abnormalities in brain regions abstractly modeling information from external data sources (Fig. 4a), but brain regions important for feedforward control were more abnormal in PCA relative to DLB (Fig. 4c). Subjects with bvFTD and SD displayed characteristic abnormalities in brain regions abstractly modeling internal data sources (Fig. 4a), but SD involved more feedforward control brain regions relative to bvFTD (Fig. 4c). Both lvPPA and dAD groups showed the most extreme abnormalities in abstract modeling brain regions relative to other dementia groups, but dAD subjects were characteristically more impaired in brain regions supporting feedback control, in line with their characteristic working memory impairment^24,29^. Typical AD is characterized by being in the middle of these extremes.

## Discussion

In our proposed computational model, neurodegeneration in dementia syndromes can be indexed using a continuous low-dimensional manifold associated with global information processing that spans the dynamic macroscale functional-anatomic organization of the brain. This model is a formulation of computational neuroscience principles focusing on ontologies relevant for clinical dysfunction in perception, cognition, and behavior. This formulation can be used to interpret our observed low-dimensional representation of the anatomy associated with the mental functions selectively impaired by neurodegenerative brain diseases that cause dementia (Figs. 2–4). The predictive ability of the model for major effects of AD on an individual (Table 2), establishes an association between our model of global functional physiology and the expression of AD within an individual. These predictive latent factors, related to information processing (Fig. 3), were decoded from patterns of glucose uptake in patients with AD, but are also able to represent meta-analytic functional activation patterns and functional connectivity gradients from cognitively normal individuals. These factors are also able to capture clinically relevant patterns of variability across seven dementia phenotypes differing them from normal aging. This construct allows for a framework for clinical reasoning based on a degenerative spectrum rather than distinct disease classes (Fig. 4). Importantly, this same manifold can be found from decoding metabolic patterns across the aging and dementia spectrum (Supplementary Figs. 5 and 6). Together, these facts lend support to computational interpretations of existing complex systems based models of neurodegenerative diseases that integrate macroscopic functional physiology with microscopic cellular and molecular physiology^5,22,24^. As this is a cross-sectional associational study design using FDG-PET as a marker of neurodegeneration, we cannot make causal predictions about brain-behavior relationships but the results here are informative for interpreting the existing literature and for hypothesis generation. These considerations are discussed in more detail below. Our study is also limited by potential cohort selection bias and generalizability to individuals not captured in our original analysis or external validation studies. We are also limited by the degree to which individual variation in FDG-PET captures degenerative biology, including technical factors such as spatial resolution, limiting the delineation of manifold dimensions potentially useful for our model.

Selective vulnerability of brain anatomy, large scale-brain networks, and the mental functions these networks and anatomy support, is a hallmark of all neurodegenerative diseases of mental function. This leads to a characteristic mapping between clinical phenotype, structural anatomy, and brain networks^12^. Our interpretation recasts these relationships in terms of degeneration in modes of brain functioning along a continuous manifold, or functional gradients. A complete model of this type of selective degeneration requires a framework for physiology that allows static brain structure to support dynamic reconfiguring of functional operations in response to current high-level demands through coordination of spiking activity in large populations of neurons across the brain globally^7^. In other words, a model bridging cognitive computational neuroscience and clinical neurology is needed. We propose that our model of neurodegeneration represents a step in that direction conceptually.

Degenerative diseases of global brain functions are an important model in which to study these proposed global neurodynamics because the pathophysiology in these conditions must selectively impair these global modes of function when they limit particular high-level functional abilities (memory, social cognition, executive control, semantic knowledge, visuospatial processing, etc.). Given the ambiguity with which the term global neurodynamics could be interpreted, we will more precisely state what is meant in this context.

Given that these brain state configurations can be represented by a low dimensional manifold in our model, such that any particular brain state can be largely characterized by a vector in this space, neurodynamics could be represented as trajectories in this state space. In other words, previously observed dynamic changes from one global brain state to another in health^5,9,11,17,48,49^ and in degenerative disease^5^ can be modeled as moving from one point in the manifold to another point representing a different global brain state^10^. Therefore, our hypothetical model suggests that aspects of degenerative diseases can be modeled as altered flow through a low dimensional functional manifold that represents large-scale network configurations related to mental functions. In this context, we simply refer to the landscape of these dynamics as the global functional state space (GFSS). Consequently, aspects of degenerative diseases of global scale mental functions can be thought of as “lesion studies” of these GFSS neurodynamics with selectively impaired functional modes, rather than damage to a functionally relevant focal brain region as is the case in structural lesion models. It is notable that our study of only AD associated cognitive impairment revealed such a manifold robust to the sample characteristics and methods used to derive it (Supplementary Figs. 2–7). This phenomenon may be explained by the wide clinical phenotypic variability in AD, the low dimensional nature of the computational manifold, and the necessary dependencies within the neurodynamics regulating the GFSS manifold.

In our analysis, three brain patterns which we relate to high-level informational processing (information source [EB-1], model type [EB-2], and control mode [EB-3]) are sufficient to explain much of the variability in degenerative pattern formation in AD and related disorders. Necessarily, these eigenbrains also encode patterns observed in the functional MRI literature. We believe this occurs because the macroscopic functional properties encoded by the manifold observed in our study index state variables of the brain’s complex adaptive information processing system at a scale relevant for high-level mental functions. These mental functions are routinely investigated in fMRI experiments and selectively degraded by neurodegenerative diseases. The proposed neurodegenerative selectivity for certain dynamic brain patterns, or modes of function of the complex information processing system, would require a fundamental role for large-scale neurodynamic physiology in AD and related disorders. This highlights the translational potential of grounding clinical neurology and cognitive psychology in terms of computational neuroscience. Our model of mental functions relevant for dementia is a step in that direction.

## Methods

### Participants

All participants or their designee provided written consent with approval of the Mayo Clinic Foundation and Olmsted Medical Center Institutional Review boards. All participants in the Mayo Clinic Rochester Alzheimer’s Disease Research Center (ADRC) and the Mayo Clinic Study of Aging (MCSA) that met our inclusion criteria were included in this study. The Mayo Clinic Rochester ADRC is a longitudinal cohort study that enrolls subjects from the clinical practice at Mayo Clinic in Rochester, MN^24^. The MCSA is a population-based study of cognitive aging among Olmsted County, MN residents^50^. Enrolled participants are adjudicated to be clinically normal or cognitively impaired by a consensus panel consisting of study coordinators, neuropsychologists, and behavioral neurologists. Methods for defining clinically unimpaired, mild cognitive impairment and dementia in both studies conform to standards in the field^51–53^. MCSA study participants receive renumeration of USD 100 as part of study participation. Both the MCSA and the ADRC studies offer assistance with ground transportation cost associated with study participation and USD 50 for participation in PET scanning portions of the study.

Inclusion criteria for this study consisted of (1) a CDR global score greater than zero, (2) presence of amyloid plaques, defined as amyloid-PET standard uptake value ratio (SUVR) >1.5, and (3) had high-quality MRI, amyloid-PET, and FDG-PET data available for analysis. A higher more conservative SUVR cut point was used for defining amyloid-PET positivity to avoid false positives^24^. See Table 1 for more details on the participants included in this study.

### Structural magnetic resonance imaging

MRI was performed on one of three compatible 3T systems from the same vendor (General Electric, Waukesha, WI, USA)^24^. A 3D magnetization prepared rapid acquisition gradient echo (MPRAGE) structural imaging sequence developed for the Alzheimer’s Disease Neuroimaging Initiative (ADNI) study was acquired^54^. All images were acquired using an 8-channel phased array head coil. Post-processing to correct for gradient distortion correction and processing has been validated in multiple studies, shown to give consistent stable results in ADNI data, and geometric fidelity after correction is independent of scanner^55,56^. Parameters were: TR/TE/T1, 2300/3/900 msec; flip angle 8°, 26 cm field of view (FOV); 256 × 256 in-plane matrix with a phase FOV of 0.94, and slice thickness of 1.2 mm. These MPRAGE parameters have been held invariant since approximately 2008. This structural MRI was used for preprocessing PET data.

### PET acquisition and preprocessing

The amyloid-PET imaging was performed with C-11 Pittsburgh Compound B^57^ and FDG-PET with F-18 fluorodeoxyglucose. PET images were acquired using 1 of 2 PET/CT scanners (DRX; GE Healthcare). A computed tomography scan was obtained for attenuation correction. These images were usually acquired on the same day with 1 h between amyloid-PET and FDG-PET acquisitions. Subjects were prepared for FDG-PET in a dimly lit room, with minimal auditory stimulation. Amyloid-PET images consisted of four 5-min dynamic frames from 40 to 60 min after injection. FDG-PET consisted of four 2-min dynamic frames acquired from 30 to 38 min after injection. PET sinograms were iteratively reconstructed into a 256 mm FOV. The pixel size was 1.0 mm and the slice thickness 3.3 mm. Standard corrections were applied.

The global amyloid-PET SUVRs were calculated as previously described^58^. The FDG-PET image volumes of each subject were coregistered to the subject’s own T1-weighted MRI scan, using a 6 degree-of-freedom affine registration with mutual information cost function. Each MRI scan was then spatially normalized to an older adult template space^59^ using a unified segmentation and normalization algorithm^60^ with transforms applied to co-registered FDG-PET images. These spatially normalized images were then intensity normalized to the pons and spatially smoothed with a 6-mm full-width half-maximum Gaussian kernel.

### Between-subject variability projection and reduction

The unsupervised machine learning framework, Between-subject variability Projection and Reduction (BPR), was designed to capture pathophysiologic information present in between-subject variability in a disease parameter of interest. The singular value decomposition (SVD) at the heart of the data reduction portion of the algorithm is widely used and interpretable, but other methods could be used depending on the framing of the problem at hand. The goals of this framework also motivate data preprocessing decisions that focus on between-subject variance within the class being studied rather than variance in the observed modality under investigation or variance relative to classes not being studied. This algorithm conceptualizes multivariate medical data from an individual as representing a particular parameterization of a (patho)physiological process of interest and uses within-class individual differences in this parametrization to define a high dimensional parameter space that contains a smaller dimensional subspace manifold that describes common features of the disease generating processes of interest. This lower dimensional subspace can be isolated in many ways, but ideally the dimensionality reduction technique used would retain interpretability in order to promote understanding of the pathophysiology of interest and be able to meaningfully place new subjects into the learned subspace and make interpretable predictions about clinical variables of interest.

In the present study, we assume that macroscale glucose uptake patterns in cognitively impaired individuals with amyloid plaque deposits represent a parameterization of macroscale AD pathophysiology. We then isolated the between-subject variability of interest to this study from these preprocessed FDG-PET scans in the following way. The preprocessed FDG-PET images are three-dimensional arrays of voxel intensities that correspond to SUVR values in a standard template space. Taking only the voxel intensities that fall within the set of voxels that have a greater than 15% probability of being gray matter in template space, this three-dimensional array can be reduced to a one-dimensional vector, **Ψ**, with *V* = 150,468 elements at our image resolution. To isolate subject effects, each element is non-parametrically standardized by the median, $\widetilde{{{{{{\bf{X}}}}}}}$, and interquartile range, $\widetilde{{{{{{\bf{Q}}}}}}}$, for that element across subjects ${{{{{{\boldsymbol{\Gamma }}}}}}}_{{{{{{\rm{i}}}}}}}=\,({{{{{\boldsymbol{\Psi }}}}}}_{{{{{{\rm{i}}}}}}}-\widetilde{{{{{{\bf{X}}}}}}}){\widetilde{{{{{{\bf{Q}}}}}}}}^{-1}$ (see Fig. 1 for surface renderings of $\widetilde{{{{{{\bf{X}}}}}}}$ and $\widetilde{{{{{{\bf{Q}}}}}}}$). Let the set of these standardized vectors, with 150,468 elements per image, be **Γ**_1_, **Γ**_2_, **Γ**_3_… **Γ**_*M*_, where *M* is the number of participants studied (*M* = 423). Subject-wise centering of each image is represented by the vector ${{{{{{\boldsymbol{\Phi }}}}}}}_{{{{{{\rm{i}}}}}}}={{{{{{\boldsymbol{\Gamma }}}}}}}_{{{{{{\rm{i}}}}}}}-\frac{1}{V}{\sum }_{{{{{{\rm{n}}}}}}=1}^{V}{{{{{{\boldsymbol{\Gamma }}}}}}}_{{{{{{\rm{i}}}}}}}$. This can then be used to represent the individual differences of interest in the brain images between each image pair, or between subject variance, by calculating the subject-wise *M* by *M* matrix **L**,

1 $${{{{{\bf{L}}}}}}=\,{{{{{{\bf{A}}}}}}}^{{{{{{\bf{T}}}}}}}{{{{{\bf{A}}}}}}$$

where the matrix **A** = [**Φ**_1_ **Φ**_2_… **Φ**_M_]. This high-dimensional projection of individual differences can be represented as an eigendecomposition, using the singular-value decomposition ${{{{{\bf{L}}}}}}={{{{{\bf{v}}}}}}{{{{{\boldsymbol{\varepsilon }}}}}}{{{{{{\bf{v}}}}}}}^{{{{{{\bf{T}}}}}}}$, such that the *M* eigenvectors, ${{{{{{\bf{v}}}}}}}_{{{{{{\bf{i}}}}}}}$_,_ of **L**, determine the linear combination of the *M* set of FDG-PET images that produce image space eigenvectors, ${{{{{{\bf{u}}}}}}}_{{{{{{\bf{l}}}}}}}$, or eigenbrains given that they can be ordered into a three-dimensional configuration corresponding to the original brain images, as previously described for the eigenfaces facial recognition algorithm for two-dimensional facial recognition^40^:

2 $${{{{{{\bf{u}}}}}}}_{l}={\sum }_{k=1}^{M}{{{{{{\bf{v}}}}}}}_{{ik}}{{{{{{\boldsymbol{\Phi }}}}}}}_{{{{{{\bf{K}}}}}}} \qquad \quad l=1,...M$$

This was demonstrated while considering that the eigenvectors ${{{{{{\bf{v}}}}}}}_{{{{{{\bf{i}}}}}}}$ of ${{{{{{\bf{A}}}}}}}^{{{{{{\bf{T}}}}}}}{{{{{\bf{A}}}}}}$ such that

3 $${{{{{{\bf{A}}}}}}}^{{{{{{\bf{T}}}}}}}{{{{{\bf{A}}}}}}{{{{{{\bf{v}}}}}}}_{{{{{{\bf{i}}}}}}}\,={{{{{{\boldsymbol{\mu }}}}}}}_{{{{{{\bf{i}}}}}}}{{{{{{\bf{v}}}}}}}_{{{{{{\bf{i}}}}}}}$$

multiplying both sides by **A**,

4 $${{{{{{\bf{AA}}}}}}}^{{{{{{\bf{T}}}}}}}{{{{{\bf{A}}}}}}{{{{{{\bf{v}}}}}}}_{{{{{{\bf{i}}}}}}}\,={{{{{{\boldsymbol{\mu }}}}}}}_{{{{{{\bf{i}}}}}}}{{{{{{\bf{Av}}}}}}}_{{{{{{\bf{i}}}}}}}$$

it is shown that ${{{{{{\bf{Av}}}}}}}_{{{{{{\bf{i}}}}}}}$ are the eigenvectors of the larger dimensional covariance matrix (150,468 by 150,468) in image space, ${{{{{\bf{C}}}}}}={{{{{\bf{A}}}}}}{{{{{{\bf{A}}}}}}}^{{{{{{\bf{T}}}}}}}$. This algorithm demonstrates how individual differences in multivariate patterns in brain images can be mapped back into the original image space in the form of a compact lower-dimensional basis-set of eigenbrains (EBs). This allows for a highly interpretable understanding of the parameterization of a disease process affecting the individuals included in the analysis.

**Fig. 5:**
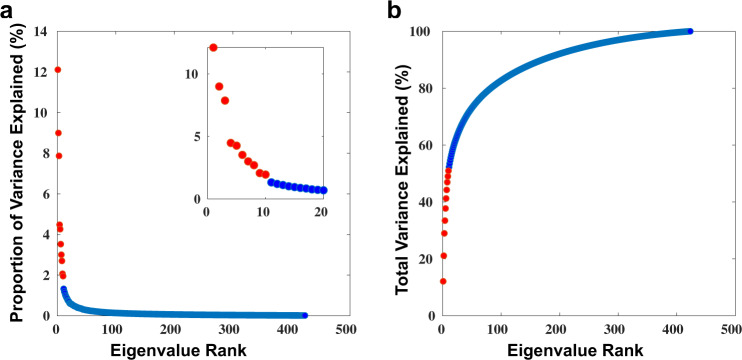
Low-rank structure. **a** A plot of the proportion of variance explained by each eigenbrain organized in rank order. The first 10 components are highlighted in red, with the remaining 413 components in blue. The same plot for the first 20 components is inset highlighting the natural break after the first 10 components. **b** A plot of the cumulative total variance explained at each rank. The majority of the variance (51%) is explained by the first 10 components that are highlighted in red with the remaining 413 components in blue. Source data are provided as a Source Data file.

The first 10 EBs (see Fig. 1 for surface renderings) explained 51% of the variance in the dataset (Fig. 5). Using only these 10 EBs, ${{{{{{\bf{u}}}}}}}_{{{{{{\bf{i}}}}}}}$, and the eigenvectors ${{{{{{\bf{v}}}}}}}_{{{{{{\bf{i}}}}}}}$, of **L**, as a subject-level weight, an individual FDG-PET scan can be estimated, ${{{{{{\boldsymbol{\Psi }}}}}}}^{{{{{{\bf{est}}}}}}},$ from a linear combination of EBs in following way:

5 $${{{{{{\boldsymbol{\Psi }}}}}}}^{{{{{{\bf{est}}}}}}}=\widetilde{{{{{{\bf{X}}}}}}}+{\sum }_{i=1}^{n=10}{{{{{{\bf{v}}}}}}}_{{{{{{\bf{i}}}}}}}{{{{{{\bf{u}}}}}}}_{{{{{{\bf{i}}}}}}}\widetilde{{{{{{\bf{Q}}}}}}}$$

An example of an estimated image using only these 10 EBs relative to the original image is presented in Supplementary Fig. 1. Using additional EBs adds additional structural information and/or individual factors, but this does not appear relevant to quantifying dysfunction in the manifold or enhance predicative ability (Supplementary Figs. 9 and 10). In addition, reconstruction with a low-rank manifold can be considered a denoising step leaving out effects of no interest (e.g., confounding structural effects seen in the red areas in the bottom of Supplementary Fig. 1b).

In order to determine the robustness of this algorithm to place an unseen image into this same manifold mapping, we iterated the algorithm 423 times leaving out each subject exactly once and estimated the subject level weights, ${{{{{{\bf{v}}}}}}}_{{{{{{\bf{i}}}}}}}$, for the left-out subject using the first 10 EBs, ${{{{{{\bf{u}}}}}}}_{{{{{{\bf{i}}}}}}}$, and the associated singular values,$\,{\varepsilon }_{i,i}$, derived from the remaining 422 subjects. These estimates were then compared to the derived values from the original run that included all 423 subjects. The set of subject-level weights, ${{{{{{\bf{v}}}}}}}_{{{{{{\rm{i}}}}}}}$, for an unseen image, ${{{{{{\boldsymbol{\Gamma }}}}}}}_{m}$, for each of the 10 EBs, ${{{{{{\bf{u}}}}}}}_{{{{{{\rm{i}}}}}}}$, was calculated in the following way:

6 $${{{{{{\boldsymbol{v}}}}}}}_{i,m}=\frac{{\sum }_{{{{{{\bf{i}}}}}}={{{{{\bf{1}}}}}}}^{{{{{{\bf{n}}}}}}={{{{{\bf{10}}}}}}}{{{{{{\boldsymbol{\Gamma }}}}}}}_{m}{{{{{{\bf{u}}}}}}}_{i}}{{{{{{{\boldsymbol{\varepsilon }}}}}}}_{{{{{{\boldsymbol{i}}}}}},{{{{{\boldsymbol{i}}}}}}}}$$

The concordance between the original values and the estimated values was assessed using the absolute value, given that the sign is indeterminate and may change on a given iteration (Supplementary Fig. 2). The method demonstrated a robust performance with Kendall’s coefficient of concordance approaching 1, indicating near complete agreement between the full model and the estimates obtained for the unseen left out subjects using Eq. (6).

To investigate the sample-related bias of the basis-set produced by this dataset, we generated 500 bootstrapped samples and calculated the first 10 EBs per sample and compared the correlation of the absolute values of the EB images produced to the EBs from the original model. All 10 EBs appeared to be robust to sample variation (Supplementary Fig. 3).

### FDG-PET eigenbrains linked to the functional organization of the brain

We used the Neurosynth database (www.neurosynth.org)^41^ and the recently described^37^ principal gradient of macroscale functional organization (available at https://neurovault.org/images/24346/) to map our FDG-PET derived EBs to patterns of functional connectivity and functional terminology. We first calculated the voxel-wise Pearson correlation between the principal gradient of functional connectivity and EB2 and found a high correlation (*r* = 0.82) (Fig. 3a). Next we compared a Neurosynth topic terms^38^ based decoding of EB2 and the principal gradient of functional connectivity. Feature terms were derived from the 50 set of topic terms (v4). Of the 50 available, 27 terms captured coherent mappings of cognitive terms spanning the theoretical range of the manifold and mirrored the range evaluated by Margulies, et al.^37^ and are used in further analysis. The decoding using all 27 topic terms is available in Table 3 for all 10 eigenbrains and the principal gradient of functional connectivity.

The decoding analysis produces a Pearson correlation between the unthresholded EB and the unthresholded topic term meta-analysis images (see the FAQs section here for details: http://neurosynth.org/decode/?neurovault=308). The topic term decoding of EB2 was similar to the same analysis performed on the principal gradient of macroscale functional organization (*r* = 0.86) in that at one extreme were regions serving concrete primary sensory/motor functions and at the other end were abstract processes involving transmodal regions (Fig. 3b). The same decoding of EB1 however revealed brain regions involved in processing external visual information were at one extreme and brain regions associated with evaluating internal mental and physical states (e.g., emotions, pain, and sustenance) were on the other extreme. EB3 was divided into brain regions involved in fluid executive control (e.g., response preparation, working memory, and response inhibition) with highly learned perceptual categories (e.g., faces, objects, and sensory perception) that can rely on feedforward control of previously learned models on the opposite extreme. The decoding weights for each of the topic terms for EB1-3 were used to associate functional terminology with the points in the three-dimensional manifold (Fig. 3c). The points in this plot were color-coded treating each EB decoding as a channel in a RGB color scheme (EB1 = Blue, inverted polarity EB2 = Red, EB3 = Green). This same RGB color-coding was done voxel-wise using the spatial loadings of EB1-3 so that a complete functional-anatomical mapping could be visualized on a brain rendering (Fig. 3d). The same color-coding is then used for the eigenvalues for individual subjects included in this study (Fig. 4).

**Fig. 6:**
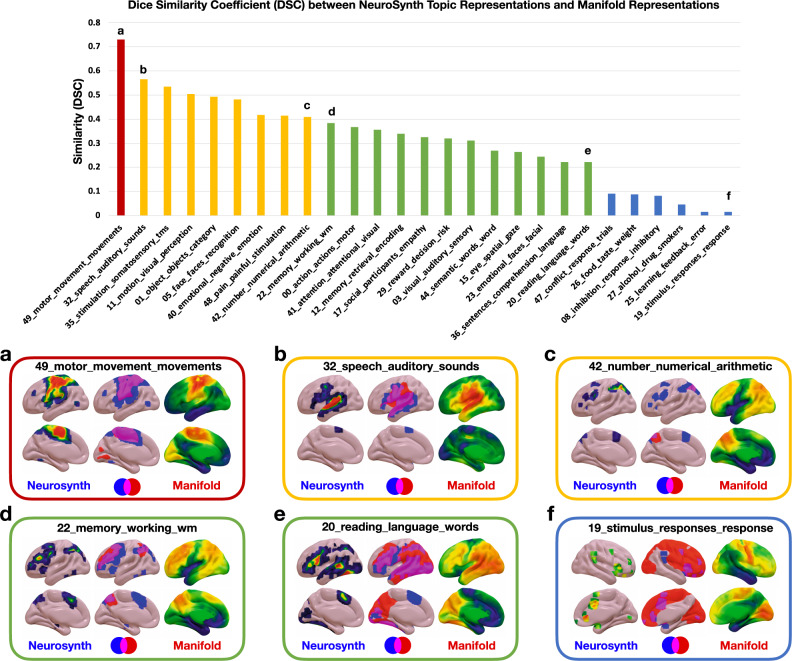
Similarity between eigenbrain representations and Neurosynth topic representations. Top, bar graph of the Dice similarity coefficient (DSC) between Neurosynth topic maps and thresholded manifold representation of topic maps order from highest to lowest DSC and colored into four similarity categories: 0-0.2 poor (blue), 0.2–0.4 fair (green), 0.4–0.6 moderate (yellow), 0.6–0.8 good (red). The letters **a**–**f** correspond to the visualization of the spatial overlap represented on surfaces below the bar graph. **a**–**f**, The thresholded Neurosynth anatomic representations, the manifold anatomic representations, and the overlap of the binarized representations (blue = Neurosynth, red = manifold, pink = overlap) are displayed on brain surfaces for these six topic terms. Source data are provided as a Source Data file.

The topic term mapping of the manifold coordinates can also be used to reconstruct the anatomic patterns associated with each functional topic (Figs. 2 and 3c). This produces a continuous representation of the anatomy associated with these topics in contrast to the discrete regions of statistically significant meta-analytic activation patterns. Thresholding the continuous manifold representations recapitulates the focal activation patterns seen in fMRI experiments summarized in the meta-analytic activation patterns (Fig. 3c). In order to quantify and better understand this phenomenon, we calculated the Dice coefficient of similarity (DSC) between the binarized topic terms (z-score threshold of 3.5 for all topics) and the binarized manifold representations at the threshold that produced the maximum DSC. The DSC is on a 0–1 scale and can be interpreted as follows: 0-0.2 poor, 0.2–0.4 fair, 0.4–0.6 moderate, 0.6–0.8 good, and 0.8-1 near complete overlap. Only 6 of the 27 topics had poor overlap, with the remainder having fair or better overlap (Fig. 6). Of these 21 topics with fair or better overlap, EB2 loading was correlated with the DSC, in contrast to having no relationship with EB1 and EB3 loadings (Supplementary Fig. 8). EB2 encodes a concrete-to-abstract functional continuum suggesting that the more abstract a cognitive function is, the more difficult it is to represent as discrete regions of activation relative to the linear combination of continuous gradients in the manifold representations.

### Statistical analysis

A combination of MATLAB (v9.4) (Mathworks Inc., Natick, MA, USA), SPM12 (https://www.fil.ion.ucl.ac.uk/spm/software/spm12/), R (v3.4.0) (http://www.R-project.org), and Cortex ID (GE Healthcare, Chicago, IL, USA) software packages were used to perform all imaging processing and statistical analyses. The Matlab Toolbox for Dimensionality Reduction was used to compare linear and non-linear techniques (https://lvdmaaten.github.io/drtoolbox/). When comparing cohort characteristics, Kruskal–Wallis one-way ANOVA was used for continuous variables and chi-squared tests were used for categorical variables. Multiple linear regression predictive models were used to for dependent variables in Table 2, the first 10 eigenvalues were used as predictors. The adjusted *R*^2^ attempts to penalize for the number of variables used in the model and is always equal to or less than the *R*^2^ value. The predicted *R*^2^ uses a leave-one-out cross-validation strategy that fits all observations but one and then predicts that left out variable with a model fit to the remainder of the observations. This procedure is repeated until each variable is left out. This value is always equal to or less than the *R*^2^ value. Large discrepancies between these values are indicative of model overfitting and poor generalizability.

### Reporting summary

Further information on research design is available in the Nature Research Reporting Summary linked to this article.

## Supplementary information

Supplementary Information

Reporting Summary

## Data Availability

The eigenimages from this study are available for download (https://neurovault.org/collections/AXJZMEAY/). A data package containing the preprocessed FDG-PET data from the AD spectrum cohort (*N* = 423) and associated data used to generate these eigenbrains from this data have been deposited in the Dryad database (10.5061/dryad.msbcc2g0n) with associated code in Zenodo (10.5281/zenodo.6030044). Data from the Mayo Clinic Study of Aging and the Mayo Clinic Alzheimer’s Disease Research Center are available upon request from these studies (https://www.mayo.edu/research/centers-programs/alzheimers-disease-research-center/data-requests). Data from the Alzheimer’s disease Neuroimaging Initiative (ADNI) and are available from the ADNI database (adni.loni.usc.edu) upon registration and compliance with the data usage agreement. Source data underlying Tables 1 and 2, Figs. 4–6, and Supplementary Figs. 2, 3, 5, 6, 8–10 are provided with this paper. Source data are provided with this paper.

## Source data

Source Data

## Code availability

A data package containing the preprocessed FDG-PET data from the AD spectrum cohort (*N* = 423) and associated data used to generate eigenbrains from this data have been deposited in the Dryad database (10.5061/dryad.msbcc2g0n) with associated code in Zenodo (10.5281/zenodo.6030044). Code used for non-linear manifold learning techniques is available from the Matlab Toolbox for Dimensionality Reduction (https://lvdmaaten.github.io/drtoolbox/).
