# Supplementary material for: A computational model of neurodegeneration in Alzheimer’s disease
Source: Nat Commun. 2022 Mar 28;13:1643. doi: 10.1038/s41467-022-29047-4 (PMC8960876; doi:10.1038/s41467-022-29047-4)
Supplement: Supplementary file 1 — Supplementary Information [file 41467_2022_29047_MOESM1_ESM.pdf]

Supplementary Information

A computational model of neurodegeneration in Alzheimer's disease

D. Jones[1,2,*], V. Lowe[2], J. Graff-Radford[1], H. Botha[1], L. Barnard[1], D. Wiepert[1], M.C. Murphy[2], M. Murray[3], M. Senjem[4], J. Gunter[4], H. Wiste[5], B. Boeve[1], D. Knopman[1], R. Petersen[1], C. Jack[2]

[1]Department of Neurology, Mayo Clinic, Rochester, MN, 55905 USA.

[2]Department of Radiology, Mayo Clinic, Rochester, MN, 55905 USA.

[3]Department of Neuroscience, Mayo Clinic, Jacksonville, FL 32224 USA.

[4]Department of Information Technology, Mayo Clinic, Rochester, MN, 55905 USA.

[5]Department of Health Sciences Research, Mayo Clinic, Rochester, MN, 55905 USA.

*Correspondence to: David T. Jones, Mayo Clinic, 200 First Street S.W., Rochester, MN 55905 jones.david@mayo.edu

SUPPLEMENTARY FIGURES

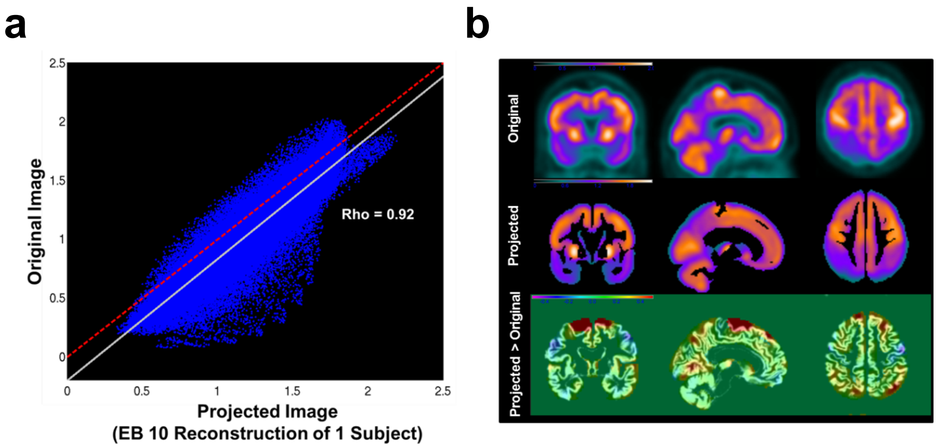

Supplementary Figure 1. **Example of reconstruction error. a** The voxel intensities in the original FDG-PET scan from one subject are plotted versus the voxel intensities of the projected images reconstructed from a 10-EB model. Red dotted line is the line of identity. The solid white line is the least square fit of the data points. **b** Orthogonal slices of the original images (top), projected image (middle), and the difference image between the two overlaid on the gray matter segmentation (bottom). The regions in red in the difference image indicates regions that are present in the projected images but are not in the original image. These regions correspond to anatomic variations in the subject's gray matter and are not related to global metabolic patterns altered by degenerative physiology.

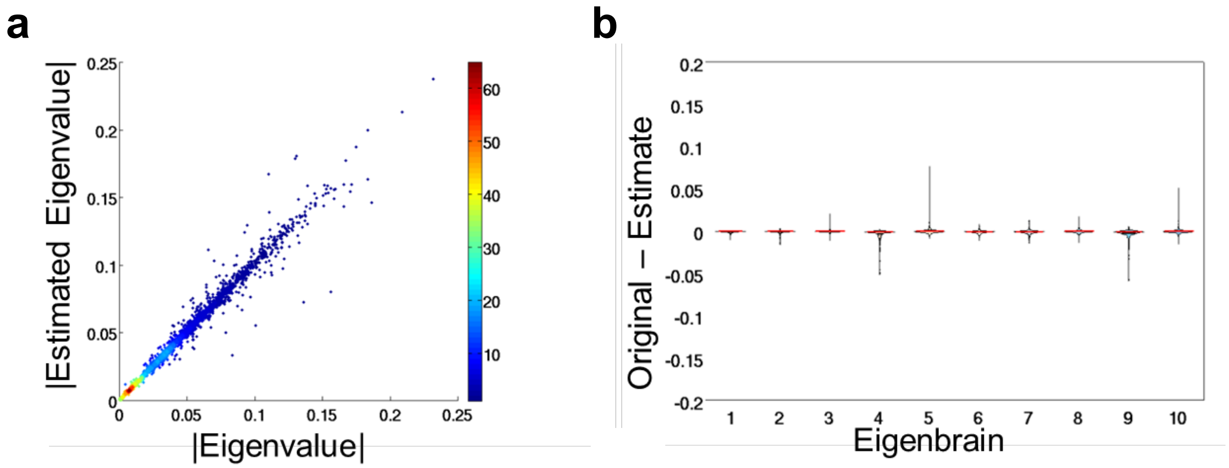

Supplementary Figure 2. **Leave-one-out cross-validation of eigenvalue estimates. A** The absolute values of the eigenvalues (n = 423 biologically independent unique patient observations with n = 10 eigenvalues per patient) estimated from a leave-one-out model are plotted versus the absolute values of the eigenvalues obtained from the full model (point density is encoded in the colormap). This shows highly consistent results for the eigenvalues whether they were calculated in the full model or estimated after leaving that subject out (Kendal's W approaching a value of 1). **B** Violin plots by eigenbrain of the difference between the eigenvalues estimated in the original full model subtracted from the values estimated during the

leave-one-out runs demonstrating similarly high reproducibility of eigenvalues across all eigenbrains. Source data are provided as a Source Data file.

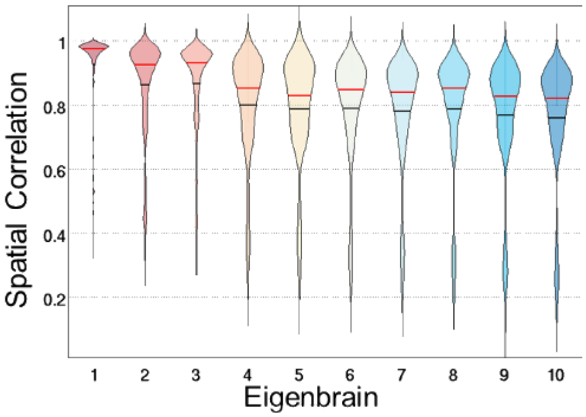

Supplementary Figure 3. **Stability of eigenbrains with bootstrapped sampling.** Violin plots of the spatial correlation between the eigenbrains estimated from the original full model and 500 bootstrapped samples (mean in black and median in red). All eigenbrains were robust to sample variation. The first three eigenbrains, that explained 29% of the variance in the original sample, were more stable than the remainder of the eigenbrains (EB4-10), that explaining 22% of the variance in the original sample. Source data are provided as a Source Data file.

SUPPLEMENTARY METHODS

Considering partial volume correction in the BPR framework

In our main analysis we did not use partial volume correction (PVC) given that we are using FDG-PET as a marker of neurodegeneration as conceptualized in the current research framework for Alzheimer's disease [1]. The rationale for using PVC on FDG-PET is to correct for low signal count in FDG-PET secondary to tissue volume differences related to several factors including, but not limited to, brain atrophy. However, atrophy is also a marker of neurodegeneration, and since that is the biological parameter of interest for the BPR analysis in this study, it would not be the correct choice to remove that signal at the same time as correcting for tissue volume that may be corrected by a low-rank representation of the data (Supplementary Fig. 1). However, when making strong claims about glucose uptake independent of tissue volume differences (not the goal of this study), this tradeoff may be desirable to answer other questions, but it is counterproductive for the BPR use case in this study. Additionally, we have found that PVC correction[2] exaggerates voxel-wise variance that confounds estimates of the between-subject covariance of interest for the BPR algorithm (vertical bands in Supplementary Fig. 4). While PVC also changes between-subject variance related to atrophy (non-uniform changes in horizontal bands in Supplementary Fig. 4), this is not a desirable property for the goals of our study. Potentially confounding global effects on subject level variation are mitigated via the subject-level mean centering before forming the between-subject covariance matrix.

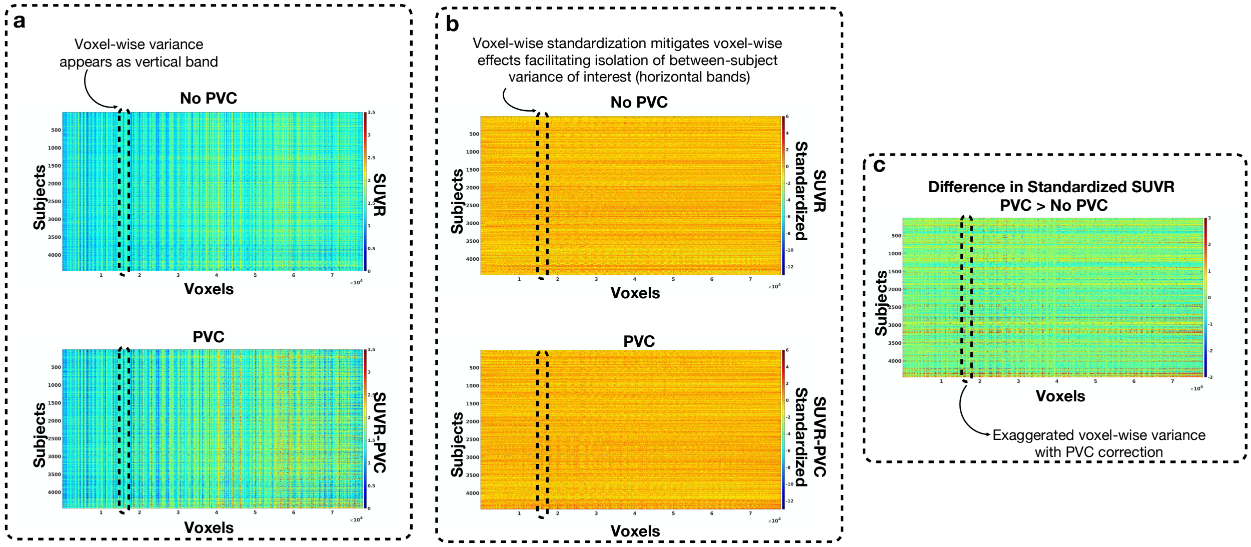

Supplementary Figure 4. **Effect of partial volume correction on between-subject variance**
a The spatially and pons intensity normalized standard uptake value ratio (SUVR) voxel-wise vectors for each subject's FDG-PET image is displayed in a subject-by-voxel matrix without (top) and with (bottom) partial volume correction (PVC). The non-standardized form of these matrices are dominated by voxel-wise variation (vertical bands). This contrasts with the standardized forms of these matrices **b** that are dominated by between-subject variance (horizontal bands). However, more voxel-wise variation is present in the standardized matrix with PVC correction **c**.

Exploring subject selection and reference cohorts in the BPR framework

We tested the effect of sample selection on the eigenbrain maps by performing the same BPR procedure on a large dataset of FDG-PET images spanning the age and degenerative disease spectrum (N = 4,448). We found that the first three eigenbrains and eigenvalues were replicated in this larger dataset, but EB1 and EB2 switched order in terms of variance explained in the larger dataset (Supplementary Fig. 5). Consistent with the BPR framework, using only a cohort of young cognitively normal participants lacking the between subject variance of interest, we were not able to replicate the eigenbrains derived from these cohorts. Similar results are found for the between-subject covariance structure in these cohorts.

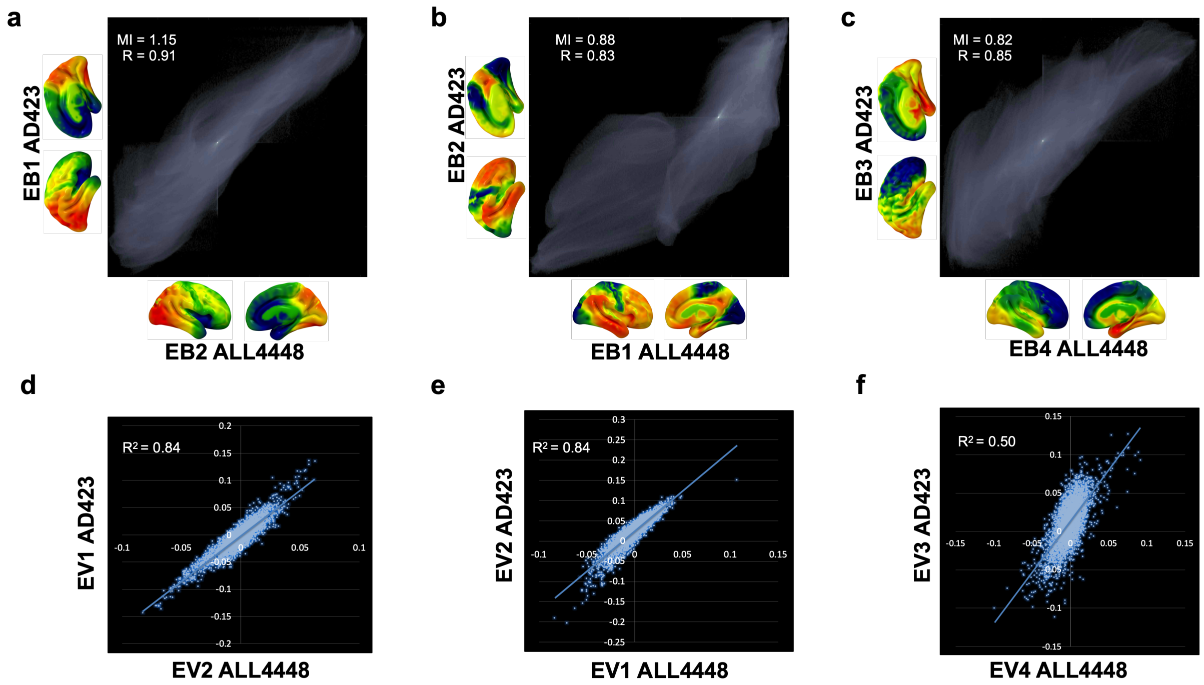

Supplementary Figure 5. **Replication of low-dimensional structure defined in the AD spectrum across a large dataset of FDG-PET images. a-c** Joint histograms between the eigenbrains defined using the AD spectrum (AD423) versus a larger FDG-PET dataset spanning the age and dementia continuum (ALL4448). The mutual information (MI) and correlation coefficient (R) is inset. **d-f** Scatter plot and linear regression for the associated eigenvalues for their respective eigenbrains. Source data are provided as a Source Data file.

We explored the between-subject covariance structure identified in our algorithm by varying the subjects used in the covariance estimation. We also test the use of out-of-sample standardization on the observed covariance structure. Although it is worth emphasizing that the goals of the BPR algorithm are to project the between-subject variance of interest defined by a particular cohort of interest hypothesized to fully parameterize a biological process of interest, and therefore out-of-sample standardization is discouraged in the BPR framework, but we directly explored these effects to emphasize this feature of the framework (Supplementary Fig. 6). The covariance structure defined by cognitively normal participants under the age of 50 (n = 91) is more dissimilar from the covariance structure defined by the entire dataset (N = 4448), cognitively normal participants across the age spectrum (n = 1765), original AD spectrum cohort (n = 423), and a random sampling of the entire dataset of the same sample size (n = 91). Standardizing to this cohort, or any out-of-sample cohort, bounds the observable between-subject covariance to the scale defined by the out-of-sample cohort. In essence, this goes against the biological motivations of BPR and limits characterizing a general manifold from a random or biological sampling of it.

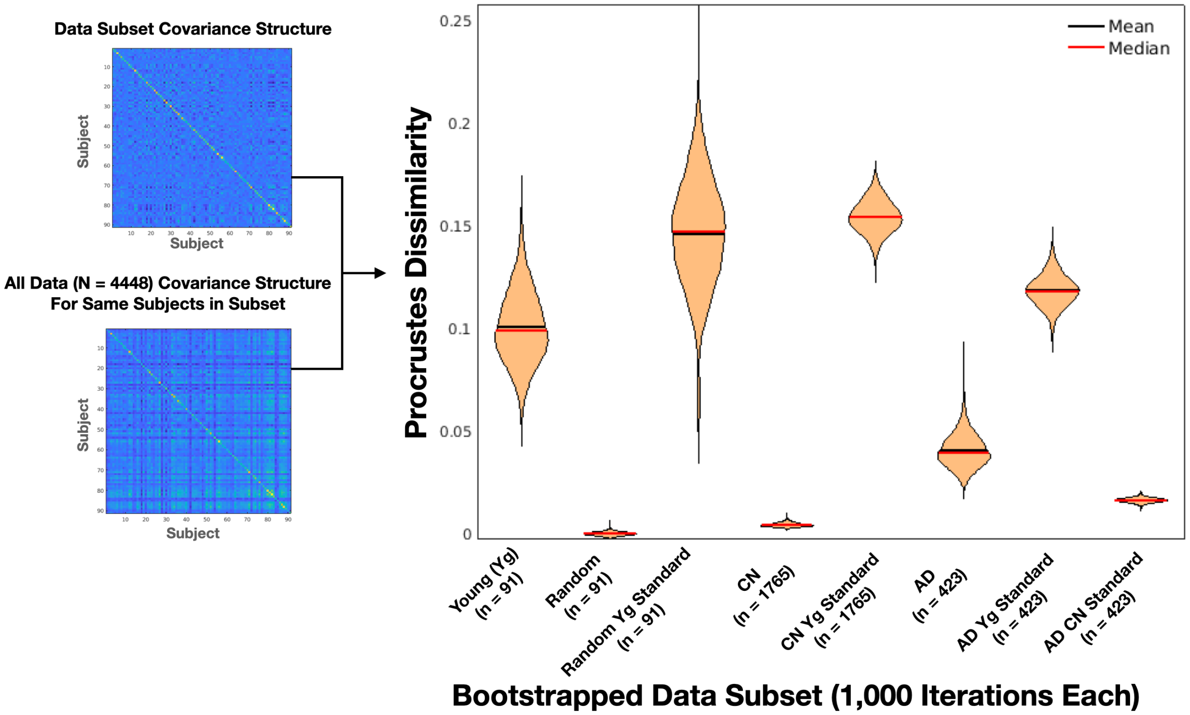

Supplementary Figure 6. **Covariance matrix dissimilarity by subsampling method.** Procrustes dissimilarity between a subset of the data put through the BPR algorithm relative to the covariance structure identified by putting all data (N = 4448) through the same algorithm. The dissimilarity observed for the same subjects between the two approaches is driven by the standardization. Each subset was subjected to 1,000 bootstrapped samples to generate distributions around point estimates for comparison (except the randomly sampled cohort, where random resampling of the 4,448 images was used). The effects of standardizing to either cognitively normal individuals under the age of 50 (Yg Standard) and the entire cognitively normal aging spectrum (CN Standard) were also compared. Source data are provided as a Source Data file.

Considering dimensionality reduction methods in the BPR framework

We explored the effect of commonly used non-linear approaches for manifold learning using dimensionality reduction, related to kernel PCA, that also do not rely on accuracy of reconstruction to define the low-rank solution, such as Laplacian eigenmaps and Isomaps. We found similar results as the linear solution provided by the standard PCA approach (Supplementary Fig. 7). This suggests that the conclusions drawn in this study would not be altered using these forms of kernel PCA for manifold learning. Given the conceptual nature of the results and potential to augment clinical reasoning and the ease of embedding new data, the highly interpretable orthogonal linear solution is preferred in this setting. This is also in concordance with previous work emphasizing the important of the orthogonality constraint that we use in the interpretation of our results.[3] However, predictive modeling, compression algorithms, and other use cases may benefit from other manifold learning approaches.

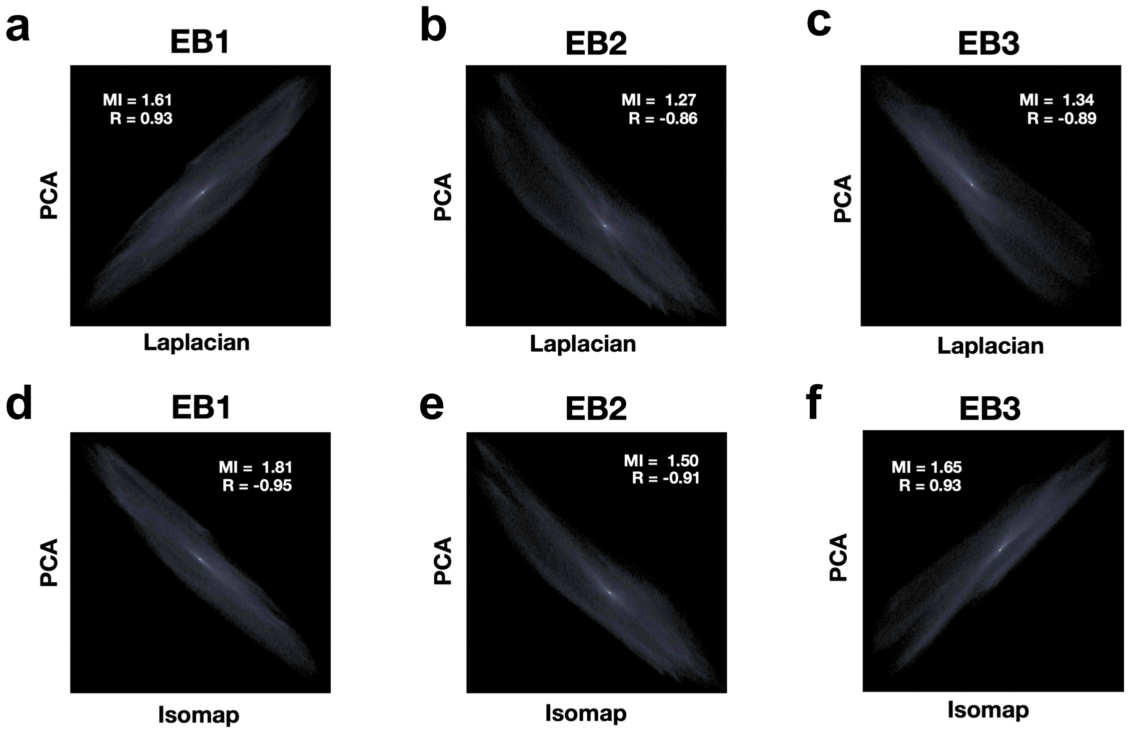

Supplementary Figure 7. **Comparing eigenbrains derived from PCA to Laplacian eigenmaps and isomaps.** Joint histograms comparing the first three eigenbrains derived from PCA to Laplacian eigenmaps **a-c** and isomaps **d-f**. All three methods produced similar manifolds with high mutual information (MI).

Eigenbrain representations and neurosynth topic representations

As described in the main text, the topic term mapping of the manifold coordinates can also be used to reconstruct the anatomic patterns associated with each functional topic. We calculated the Dice coefficient of similarity (DSC) between the binarized topic terms (z-score threshold of 3.5 for all topics) and the binarized manifold representations at the threshold that produced the maximum DSC. The DSC is on a 0-1 scale and can be interpreted as follows: 0-0.2 poor, 0.2–0.4 fair, 0.4–0.6 moderate, 0.6–0.8 good, and 0.8-1 near complete overlap. Only 6 of the 27 topics had poor overlap, with the remainder having fair or better overlap (Fig. 6). Of these 21 topics with fair or better overlap, EB2 loading was correlated with the DSC, in contrast to having no relationship with EB1 and EB3 loadings (Supplementary Fig. 8). EB2 encodes a concrete-to-abstract functional continuum suggesting that the more abstract a cognitive function is, the more difficult it is to represent as discrete regions of activation relative to the linear combination of continuous gradients in the GFSS representations.

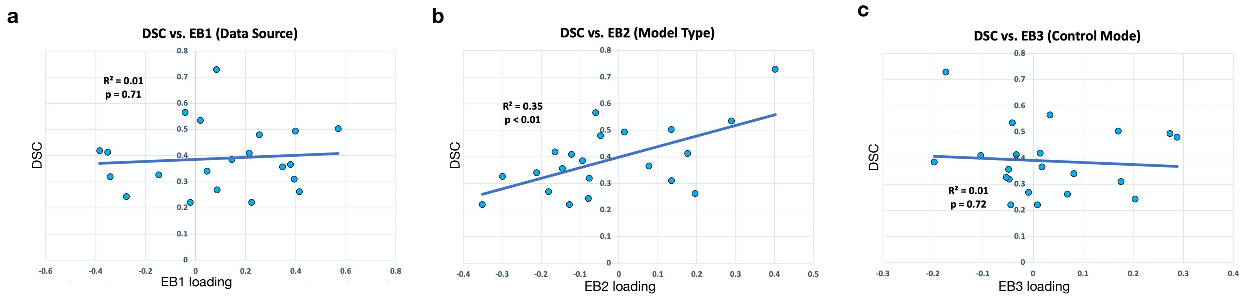

Supplementary Figure 8. **Manifold embedding varies by model type of cognitive function.** Greater similarity between GFSS representations and neurosynth topic representations is associated with more concrete cognitive functions. Scatter plot of the Dice similarity coefficient (DSC) between neurosyth topic maps and thresholded GFSS topic maps and **a** EB1, **b** EB2, and **c** EB3 loadings for each topic. The linear regression line, R^2, and two-sided p-value are displayed for each without adjustments for multiple comparisons. Source data are provided as a Source Data file.

Out-of-sample predictions in ADNI

To evaluate the out-of-sample predictive ability of the manifold for key measures of the effects of AD pathophysiology (i.e., age, glucose uptake, cognition, and disease severity), we used the publicly available multisite data from the Alzheimer's Disease Neuroimaging Initiative (ADNI) database (adni.loni.usc.edu). This multisite database is well suited for testing the ability of the manifold to make predictions about individual subjects with biomarker evidence of AD pathophysiology. The focus of this validation analysis was on predicting an individual's age based on their metabolic mapping to state space. Age prediction is a strong validation of the manifold mapping given the association between age and clinical phenotypes [4], pathologic phenotypes [5], and global tau deposition patterns [6]. We analyzed the data from 410 ADNI subjects (Table 1) with FDG-PET scans, CDR greater than 0, and positive amyloid PET imaging as defined by previously established ADNI cut-point (1.11 for whole cerebellum referenced AV45 data) [7].

In this dataset, the FDG composite summary used in ADNI to summarize AD-like patterns of hypometabolism [8] is not associated with age (Supplementary Fig. 9a). However, once the eigenvalues for each of the first 10 EBs are calculated from an individual's FDG scan, the manifold model fits in the Mayo data (Table 2) can be used to accurately predict the age of an individual (Supplementary Fig. 9b) and the FDG composite score (Supplementary Fig. 9c). The predictive ability of the manifold models in the ADNI data is not substantially improved with additional EBs being included in the models for FDG composite, age, disease severity, or cognition (Supplementary Fig. 10).

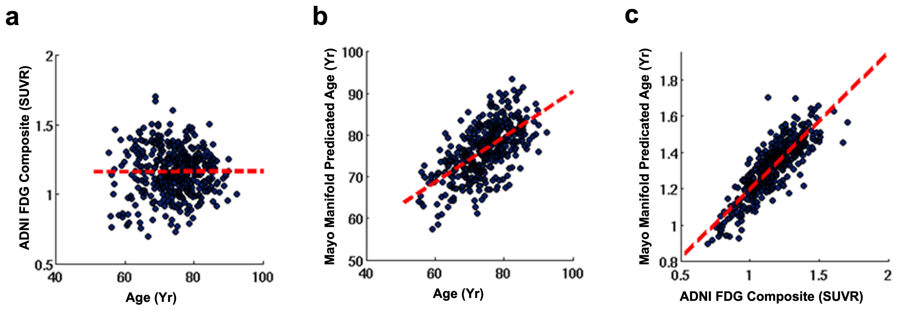

Supplementary Figure 9. **Manifold predictive modeling.** **a** The ADNI FDG composite score is not associated with age. **b** Using the FDG-PET to place the subjects into the 10-dimensional manifold allows for accurate prediction of the subjects age using models fits from the Mayo data (Table 2). **c** The manifold embedding also allows for an accurate prediction of the subjects FDG-PET composite score. See Supplementary Fig. 10 for a range of correlation values by number of eigenbrains used in the predictive model. Source data are provided as a Source Data file.

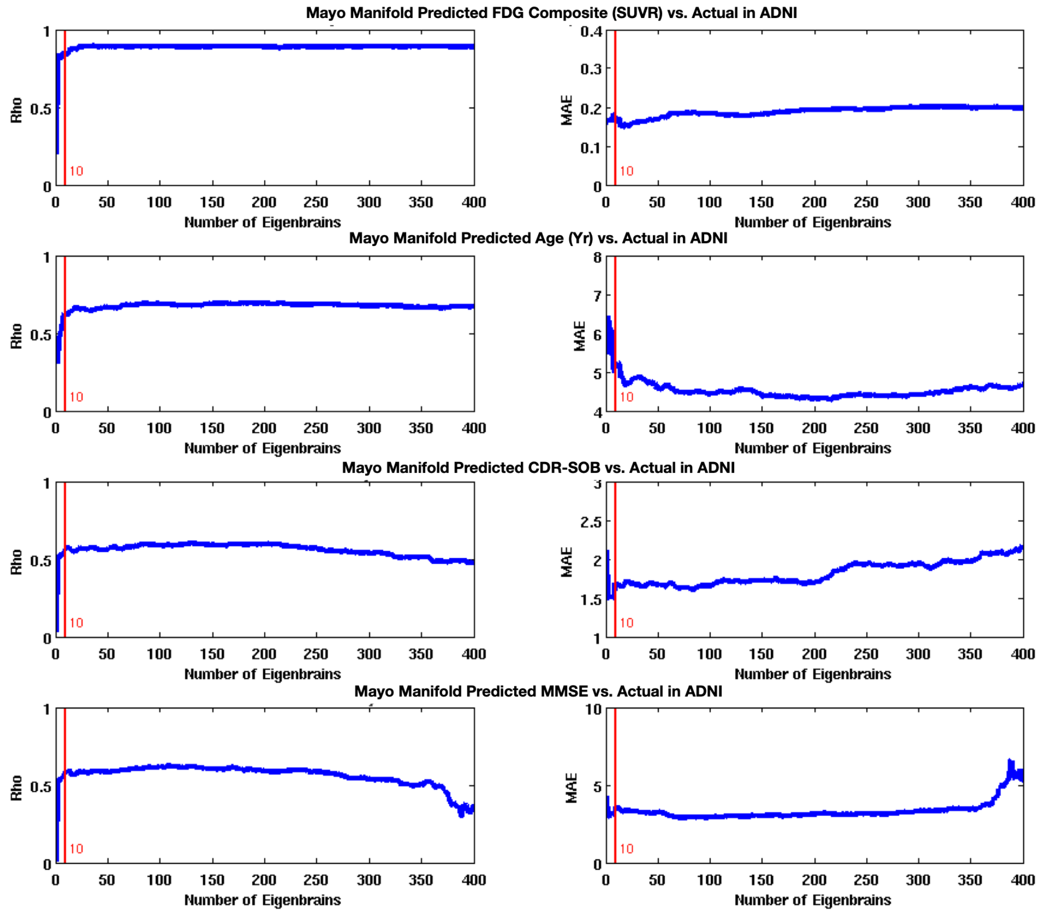

Supplementary Figure 10. Plots of model order effect on manifold predictive modeling of FDG, age, severity, and cognition in ADNI. The Pearson correlation coefficients between the actual and predicted values are plotted versus the number of eigenbrains used in the predictive model for each of the variables (left). The mean absolute error (MAE) of the prediction is plotted versus the number of eigenbrains used in the predictive model for each of the variables (right). The near optimal performance of the 10-D manifold model is highlighted by the red vertical bar in each plot. Source data are provided as a Source Data file.

SUPPLEMENTARY REFERENCES

1. Jack CR, Jr.*, et al.* NIA-AA Research Framework: Toward a biological definition of Alzheimer's disease. *Alzheimers Dement* **14**, 535-562 (2018).

2. Meltzer CC*, et al.* Comparative evaluation of MR-based partial-volume correction schemes for PET. *J Nucl Med* **40**, 2053-2065 (1999).

3. Shine JM*, et al.* Human cognition involves the dynamic integration of neural activity and neuromodulatory systems. *Nat Neurosci* **22**, 289-296 (2019).

4. Barnes J, Dickerson BC, Frost C, Jiskoot LC, Wolk D, van der Flier WM. Alzheimer's disease first symptoms are age dependent: Evidence from the NACC dataset. *Alzheimers Dement* **11**, 1349-1357 (2015).

5. Murray ME, Graff-Radford NR, Ross OA, Petersen RC, Duara R, Dickson DW. Neuropathologically defined subtypes of Alzheimer's disease with distinct clinical characteristics: a retrospective study. *Lancet Neurol* **10**, 785-796 (2011).

6. Jones DT*, et al.* Tau, amyloid, and cascading network failure across the Alzheimer's disease spectrum. *Cortex* **97**, 143-159 (2017).

7. Joshi AD*, et al.* Performance characteristics of amyloid PET with florbetapir F 18 in patients with alzheimer's disease and cognitively normal subjects. *J Nucl Med* **53**, 378-384 (2012).

8. Landau SM*, et al.* Associations between cognitive, functional, and FDG-PET measures of decline in AD and MCI. *Neurobiol Aging* **32**, 1207-1218 (2011).
