# Supplementary material for: A computational model of neurodegeneration in Alzheimer’s disease
Source: Nat Commun. 2022 Mar 28;13:1643. doi: 10.1038/s41467-022-29047-4 (PMC8960876; doi:10.1038/s41467-022-29047-4)
Supplement: Supplementary file 2 — Reporting Summary [file 41467_2022_29047_MOESM2_ESM.pdf]

nature research

Corresponding author(s): David Jones

Last updated by author(s): Jan 25, 2022

Reporting Summary

Nature Research wishes to improve the reproducibility of the work that we publish. This form provides structure for consistency and transparency in reporting. For further information on Nature Research policies, see our Editorial Policies and the Editorial Policy Checklist.

Statistics

For all statistical analyses, confirm that the following items are present in the figure legend, table legend, main text, or Methods section.

n/a	Confirmed	
☐	☒	The exact sample size (*n*) for each experimental group/condition, given as a discrete number and unit of measurement
☐	☒	A statement on whether measurements were taken from distinct samples or whether the same sample was measured repeatedly
☐	☒	The statistical test(s) used AND whether they are one- or two-sided<br>*Only common tests should be described solely by name; describe more complex techniques in the Methods section.*
☐	☒	A description of all covariates tested
☐	☒	A description of any assumptions or corrections, such as tests of normality and adjustment for multiple comparisons
☐	☒	A full description of the statistical parameters including central tendency (e.g. means) or other basic estimates (e.g. regression coefficient) AND variation (e.g. standard deviation) or associated estimates of uncertainty (e.g. confidence intervals)
☐	☒	For null hypothesis testing, the test statistic (e.g. *F*, *t*, *r*) with confidence intervals, effect sizes, degrees of freedom and *P* value noted<br>*Give P values as exact values whenever suitable.*
☒	☐	For Bayesian analysis, information on the choice of priors and Markov chain Monte Carlo settings
☒	☐	For hierarchical and complex designs, identification of the appropriate level for tests and full reporting of outcomes
☒	☐	Estimates of effect sizes (e.g. Cohen's *d*, Pearson's *r*), indicating how they were calculated

Our web collection on statistics for biologists contains articles on many of the points above.

Software and code

Policy information about availability of computer code

Data collection	PET images were acquired using 1 of 2 PET/CT scanners (DRX; GE Healthcare, Waukesha, WI, USA). MRI was performed on one of three compatible 3T systems from the same vendor (General Electric, Waukesha, WI, USA).
Data analysis	A combination of MATLAB (v9.4) (Mathworks Inc., Natick, MA, USA), SPM12 (https://www.fil.ion.ucl.ac.uk/spm/software/spm12/) , R (v3.4.0) (http://www.R-project.org), and CortexID (GE Healthcare, Chicago, IL, USA) software packages were used to perform all imaging processing and statistical analyses. The Matlab Toolbox for Dimensionality Reduction was used to compare linear and non-linear techniques (https://lvdmaaten.github.io/drtoolbox/). NeuroSynth (www.neurosynth.org) was used for meta-analytic topic terms analysis. A data and code package containing the preprocessed FDG-PET data from the AD spectrum cohort (N = 423) and code used to generate eigenbrains from this data have been deposited in the Dryad database (https://doi.org/10.5061/dryad.msbcc2g0n).

For manuscripts utilizing custom algorithms or software that are central to the research but not yet described in published literature, software must be made available to editors and reviewers. We strongly encourage code deposition in a community repository (e.g. GitHub). See the Nature Research guidelines for submitting code & software for further information.

Data

Policy information about availability of data

All manuscripts must include a data availability statement. This statement should provide the following information, where applicable:

- Accession codes, unique identifiers, or web links for publicly available datasets
- A list of figures that have associated raw data
- A description of any restrictions on data availability

The eigenimages from this study are available for download (https://neurovault.org/collections/AXJZMEAY/). A data and code package containing the preprocessed FDG-PET data from the AD spectrum cohort (N = 423) and code used to generate these eigenbrains from this data have been deposited in the Dryad database

(https://doi.org/10.5061/dryad.msbcc2g0n). Data from the Mayo Clinic Study of Aging and the Mayo Clinic Alzheimer's Disease Research Center are available upon request from these studies (https://www.mayo.edu/research/centers-programs/alzheimers-disease-research-center/data-requests). Data from the Alzheimer's disease Neuroimaging Initiative (ADNI) and are available from the ADNI database (adni.loni.usc.edu) upon registration and compliance with the data usage agreement. Source data underlying Tables 1, 2, Figs. 4, 5, 6 and Supplementary Figures 2, 3, 5, 6, 8, 9, and 10 are provided with this paper.

Field-specific reporting

Please select the one below that is the best fit for your research. If you are not sure, read the appropriate sections before making your selection.

☒ Life sciences ☐ Behavioural & social sciences ☐ Ecological, evolutionary & environmental sciences

For a reference copy of the document with all sections, see nature.com/documents/nr-reporting-summary-flat.pdf

Life sciences study design

All studies must disclose on these points even when the disclosure is negative.

Sample size	We included data from all available participants that met our inclusion criteria. We explored the effects of sampling in several experiments detailed in our main text and response document.
Data exclusions	None based on inclusion criteria of quality controlled database.
Replication	Bootstrapped sampling of the 423 AD spectrum cohort, leave-one-out cross-validation of the 423 AD-spectrum cohort, and out-of-sample replication including all available FDG-PET data (N = 4448) and the Alzheimer's Diseases Neuroimaging Initiative (ADNI) cohort. These experiments were conducted once as part of this study and were successful as reported in the main text and Supplementary Information. The source data for these studies is available in the Source Data file.
Randomization	Observational cohort study.
Blinding	Observational cohort study.

Reporting for specific materials, systems and methods

We require information from authors about some types of materials, experimental systems and methods used in many studies. Here, indicate whether each material, system or method listed is relevant to your study. If you are not sure if a list item applies to your research, read the appropriate section before selecting a response.

Materials & experimental systems

n/a	Involved in the study	
☒	☐	Antibodies
☒	☐	Eukaryotic cell lines
☒	☐	Palaeontology and archaeology
☒	☐	Animals and other organisms
☐	☒	Human research participants
☒	☐	Clinical data
☒	☐	Dual use research of concern

Methods

n/a	Involved in the study	
☒	☐	ChIP-seq
☒	☐	Flow cytometry
☐	☒	MRI-based neuroimaging

Human research participants

Policy information about studies involving human research participants

Population characteristics	See Table 1.
Recruitment	All participants in the Mayo Clinic Rochester Alzheimer's Disease Research Center and the Mayo Clinic Study of Aging that met our inclusion criteria were included in this study. The Mayo Clinic Rochester Alzheimer's Disease Research Center is a longitudinal cohort study that enrolls subjects from the clinical practice at Mayo Clinic in Rochester, MN. The Mayo Clinic Study of Aging is a population-based study of cognitive aging among Olmsted County, MN residents. Enrolled participants are adjudicated to be clinically normal or cognitively impaired by a consensus panel consisting of study coordinators, neuropsychologists and behavioral neurologists. Methods for defining clinically unimpaired, mild cognitive impairment and dementia in both of these studies conform to standards in the field. Given the ADRC cohort is recruited from the clinical practice in Rochester, MN there is referral bias and other forms of selection bias, information bias, and confounding. While selection bias is minimized via population based sampling in the Mayo Clinic Study of Aging, some element of this and other cohort bias will remain. There is also poor generalizability of cohort studies to individuals not captured in the cohort.
Ethics oversight	All participants or their designee provided written consent with approval of the Mayo Clinic Foundation and Olmsted Medical

Ethics oversight	Center Institutional Review boards.

Note that full information on the approval of the study protocol must also be provided in the manuscript.

Magnetic resonance imaging

Experimental design

Design type	Resting state
Design specifications	1 scan per subject
Behavioral performance measures	Resting state

Acquisition

Imaging type(s)	Structural MRI and FDG-PET
Field strength	3T
Sequence & imaging parameters	Structural Magnetic Resonance Imaging<br>MRI was performed on one of three compatible 3T systems from the same vendor (General Electric, Waukesha, WI, USA). A 3D magnetization prepared rapid acquisition gradient echo (MPRAGE) structural imaging sequence developed for the Alzheimer's Disease Neuroimaging Initiative (ADNI) study was acquired. All images were acquired using an 8-channel phased array head coil. Post-processing to correct for gradient distortion correction and processing has been validated in multiple studies, shown to give consistent stable results in ADNI data, and geometric fidelity after correction is independent of scanner 51,52. Parameters were: TR/TE/T1, 2300/3/900 msec; flip angle 8°, 26 cm field of view (FOV); 256 × 256 in-plane matrix with a phase FOV of .94, and slice thickness of 1.2 mm. These MPRAGE parameters have been held invariant since approximately 2008. This structural MRI was used for preprocessing PET data.<br><br>PET Acquisition and Preprocessing<br>The amyloid-PET imaging was performed with C-11 Pittsburgh Compound B 53 and FDG-PET with F-18 fluorodeoxyglucose. PET images were acquired using 1 of 2 PET/CT scanners (DRX; GE Healthcare). A computed tomography scan was obtained for attenuation correction. These images were usually acquired on the same day with 1 hour between amyloid-PET and FDG-PET acquisitions. Subjects were prepared for FDG-PET in a dimly lit room, with minimal auditory stimulation. Amyloid-PET images consisted of four 5-min dynamic frames from 40 to 60 min after injection. FDG-PET consisted of four 2-min dynamic frames acquired from 30 to 38 min after injection. PET sinograms were iteratively reconstructed into a 256 mm FOV. The pixel size was 1.0 mm and the slice thickness 3.3 mm. Standard corrections were applied.
Area of acquisition	Whole brain.

Diffusion MRI ☐ Used ☒ Not used

Preprocessing

Preprocessing software	Matlab v9.4<br>Statistics and Machine Learning Toolbox v11.3<br>Parallel Computing Toolbox v6.12<br>MATLAB Distributed Computing Server v6.12<br>Jimmy Shen (2021). Tools for NIfTI and ANALYZE image (https://www.mathworks.com/matlabcentral/fileexchange/8797-tools-for-nifti-and-analyze-image), MATLAB Central File Exchange. Retrieved April 28, 2021.<br>SPM12 (https://www.fil.ion.ucl.ac.uk/spm/software/spm12/)
Normalization	The global amyloid-PET SUVRs were calculated as previously described (30). The FDG-PET image volumes of each subject were coregistered to the subject's own T1-weighted MRI scan, using a 6 degree-of-freedom affine registration with mutual information cost function. Each MRI scan was then spatially normalized to an older adult template space using a unified segmentation and normalization algorithm with transforms applied to co-registered FDG-PET images. These spatially normalized images were then intensity normalized to the pons and spatially smoothed with a 6 mm full-width half-maximum Gaussian kernel.
Normalization template	Each MRI scan was then spatially normalized to an older adult template space using a unified segmentation and normalization algorithm with transforms applied to co-registered FDG-PET images.
Noise and artifact removal	These spatially normalized images were then intensity normalized to the pons and spatially smoothed with a 6 mm full-width half-maximum Gaussian kernel.
Volume censoring	No censoring

Statistical modeling & inference

Model type and settings: No task

Effect(s) tested: No task

Specify type of analysis: ☒ Whole brain ☐ ROI-based ☐ Both

Statistic type for inference (See Eklund et al. 2016): No voxel- or cluster-wise statistics

Correction: No voxel- or cluster-wise statistics

Models & analysis

n/a	Involved in the study
☒	☐ Functional and/or effective connectivity
☒	☐ Graph analysis
☐	☒ Multivariate modeling or predictive analysis

Multivariate modeling and predictive analysis: For each of the dependent variables in Table 2, the first 10 eigenvalues were used as predictors in a multivariate linear regression model. The number of subjects included, R2, adjusted R2, predicted R2, standardized beta coefficients, p-value, and Bonferroni adjusted p-values are displayed for each model. Using the simple multivariate linear regression models from this cohort (Table 2) to predict the age of patients from an independent database (N = 410) available as part of the Alzheimer's Disease Neuroimaging Initiative (Table 1), we achieved a mean absolute error of 5.1 years using a linear 10 EB model. Similar results were obtained predicting other variables in the dataset related to glucose uptake, cognition, and disease severity, with peak prediction performance achieved with models using 8-20 EBs.
